# Bounding the efficiency gain of differentiable road pricing for EVs and GVs to manage congestion and emissions

**DOI:** 10.1371/journal.pone.0234204

**Published:** 2020-07-30

**Authors:** Haoning Xi, Liu He, Yi Zhang, Zhen Wang

**Affiliations:** 1 Research Center for Integrated Transport Innovation (RCITI), School of Civil and Environmental Engineering, University of New South Wales, Sydney, New South Wales, Australia; 2 Department of Automation, Nanjing University of Science and Technology, Nanjing, Jiangsu, China; 3 Tsinghua-Berkeley Shenzhen Institute, Tsinghua University, Shenzhen, PR China; 4 Tsinghua Shenzhen International Graduate School, Tsinghua University, Shenzhen, PR China; 5 UBTECH Sydney Artificial Intelligence Centre, School of Computer Science, The University of Sydney, New South Wales, Australia; Shandong University of Science and Technology, CHINA

## Abstract

Increasing concerns about air pollution and the promise of enhancing energy security have stimulated the growth of electric vehicles (EVs) worldwide. Compared with gasoline vehicles (GVs), EVs have no emissions and are more environmentally friendly to the sustainable transportation system. Since these two types of vehicles with different emission externalities and observable differences, in this paper, we propose a differentiable road pricing for EVs and GVs to simultaneously manage congestion and emissions by establishing a two-class bi-objective optimization (TCBO) model. First, we investigate whether the differentiable road pricing can induce user equilibrium pattern into a unique pareto-efficient pattern. Then performance of the bi-criteria system optimal is measured by bounding the deviation gap of the Pareto frontier. Specifically, we bound how far the total system travel time and total system emissions at a given Pareto optimum can deviate from their respective single-criterion based system optimum. Finally, we investigate the maximum efficiency gain of the bi-criteria system achieved through implementing differentiable road pricing by comparing total system travel time and total system emissions under two states. After defining two types of price of anarchy (POA), the theoretical bound for the worst possible ratio of total system travel time\ total system emissions in user equilibrium state to the total system travel time\ total system emissions in Pareto-efficient state is derived out. In order to validate the feasibility of theoretical bound, we conduct case studies to calculate the numerical bound of POA based on two Chinese cities: Shenzhen and Lasa. Overall, quantifying the maximum efficiency of differentiable road pricing is beneficial for improving the network designing, policy implementation and social efficiency with regard to congestion and emissions caused by EV and GV users.

## 1. Introduction

Selfish travelers usually pursue their own optimal strategies and will not achieve the social optimum, which has led to great losses for the whole society. It is shown that the system optimum can be decentralized as a user equilibrium by charging the optimal toll on each link, which is equal to the marginal social cost and marginal private cost [1–2, 3]. Since Pigou [4] first introduced marginal-cost pricing, it has been one of the most efficient ways to manage traffic demand and has been a long-standing research topic. The existing literature has investigated marginal cost pricing in multiclass traffic assignment. Netter [5] and Dafermos and Sparrow [6] discussed marginal cost pricing for different vehicle types, such as trucks, passenger cars and buses, in terms of vehicle size. Moreover, enhanced awareness of environmental protection has led to the wide adoption of ecofriendly electric vehicles all over the world. Global EV sales exceeded 5.1 million in 2018, which was almost double the number of new electric car sales in 2017 [7]. The International Energy Agency (IEA) forecasts that the number of electric vehicles on the road around the world will hit 125 million by 2030 [8]. Since EVs and GVs can be physically distinguished and have different impacts on total system emissions, we should consider differentiable marginal social pricing for EVs and GVs.

Furthermore, the multiclass problem has been extended into multiclass multicriteria problems by integrating travel time cost and monetary cost simultaneously. For example, Yang and Huang [9] and Guo and Yang [10] proved the existence of valid tolls that can decentralize multiclass multicriteria network equilibrium flow patterns when travelers value time differently, and the system cost can be measured in terms of either time or monetary units.

Environmental issues and sustainable transportation have attracted ever-increasing attention worldwide in recent years. Congestion and emissions are two types of disutility that negatively impact our daily life in various ways, from being a minor health inconvenience to affecting job satisfaction. An increasing number of studies consider both congestion and emissions as two aspects of traffic assignment problems. Yin [11] that emission-oriented road pricing for single class users can be used to induce the flow pattern with minimum emissions and established a bi-objective optimization model to identify the Pareto optimal solutions for total travel time and total emissions. Wen [12] proposed a bilevel pricing model to minimize emissions and the total travel time based on the dynamic user equilibrium. Wang [13] reviewed the development of dynamic traffic assignment models considering emission road pricing. Chen and Yang [14] established the necessary and sufficient conditions for road pricing to decentralize a Pareto-efficient flow pattern in terms of congestion and emissions.

The methods used to bound the efficiency gain of a pricing scheme have been proven to be very important and meaningful for evaluating pricing schemes. The upper bound of the ratio of the social welfare between the competitive equilibrium and the social optimum is established to quantify the inefficiency of the oligopolistic equilibria via higher tolls and less demand [15]. The efficiency loss of a general second-best pricing scheme is obtained by bounding a given road pricing scheme [16]. The upper bound of the User equilibrium-Cournot–Nash (UE-CN) mixed equilibrium traffic assignment is derived by analytic derivation when road pricing is considered (not considered) part of the total travel costs [17].

The efficiency gain of differentiable road pricing can be bounded by considering the notion of the price of anarchy (POA), which is a concept used in economics and computer science to qualify the inefficiency of the Nash equilibrium by seeking the worst possible ratio between the total cost incurred by untolled travelers and the system optimum in terms of congestion and emissions under differentiable tolls. After Koutsoupias and Papadimitriou [18] proposed the concept of the price of anarchy, Roughgarden [19] proved that the price of anarchy is independent of the network topology when the cost function is linear and nonlinear.

Research on qualifying the POA under different types of cost functions can be classified into two types. The first type is related to the theoretical bound of the POA and includes a series of studies, such as [18–24]. Correa et al. [25] proved the bound of POA through a geometric method, and the results are consistent with those in [24]. By introducing valid tolls, Karakostas and Kolliopoulos [26] indicated that POA decreases when link time functions are continuous and strictly increasing. Investigating a multiclass multicriteria problem, Han and Yang [27] derived the bound of the POA in different cases: the tolls were both considered and not considered as a system cost under the time criterion and cost criterion functions, respectively. The second type is related to the numerical bound of the POA, which can be calculated by considering large-scale networks. Youn *et al*. [28] estimated the POA by analyzing the road networks of Boston, London and New York, and the simulation results showed that this value can reach 1.3. Grange *et al*. [29] provided the results of case studies on the POA in three networks, considering the differences between the demand levels of each network. Liao *et al*. [30] carried out a simulation and calculated the value of the POA based on the Sioux Falls network. O’Hare et al. [31] proposed four types of mechanisms that explain the relationship between the POA and travel demand in urban networks when the link delay functions are increasing and separable. Zhang et al. [32] carried out a case study and calculated the value of the POA using actual road network traffic data from Eastern Massachusetts.

In summary, road pricing has been considered in the context of a multicriteria (time vs cost) equilibrium, different vehicle types in terms of size, and a bicriteria (congestion and emission) system optimum. However, to the best of our knowledge, no attempt has been made to look into differentiable road pricing for EVs and GVs to simultaneously manage congestion and emissions. Moreover, the existing literature has never qualified the efficiency gain of such differentiable road pricing for EVs and GVs in terms of congestion and emissions. To fill these gaps, this paper will propose differentiable road pricing for EVs and GVs by establishing a two-class bi-objective optimization (TCBO) model to simultaneously manage congestion and emissions. Since EVs and GVs can be physically distinguished, differentiable road pricing for EVs and GVs is feasible. After investigating the decentralization of differentiable road pricing, the theoretical upper bound of *POA*_*T*_ will be derived to qualify the efficiency gain of differentiable road pricing in terms of system total travel time. Finally, we report the numerical bounds of *POA*_*T*_ and *POA*_*E*_ by conducting simulations based on the actual networks in Shenzhen.

**Fig 1:**
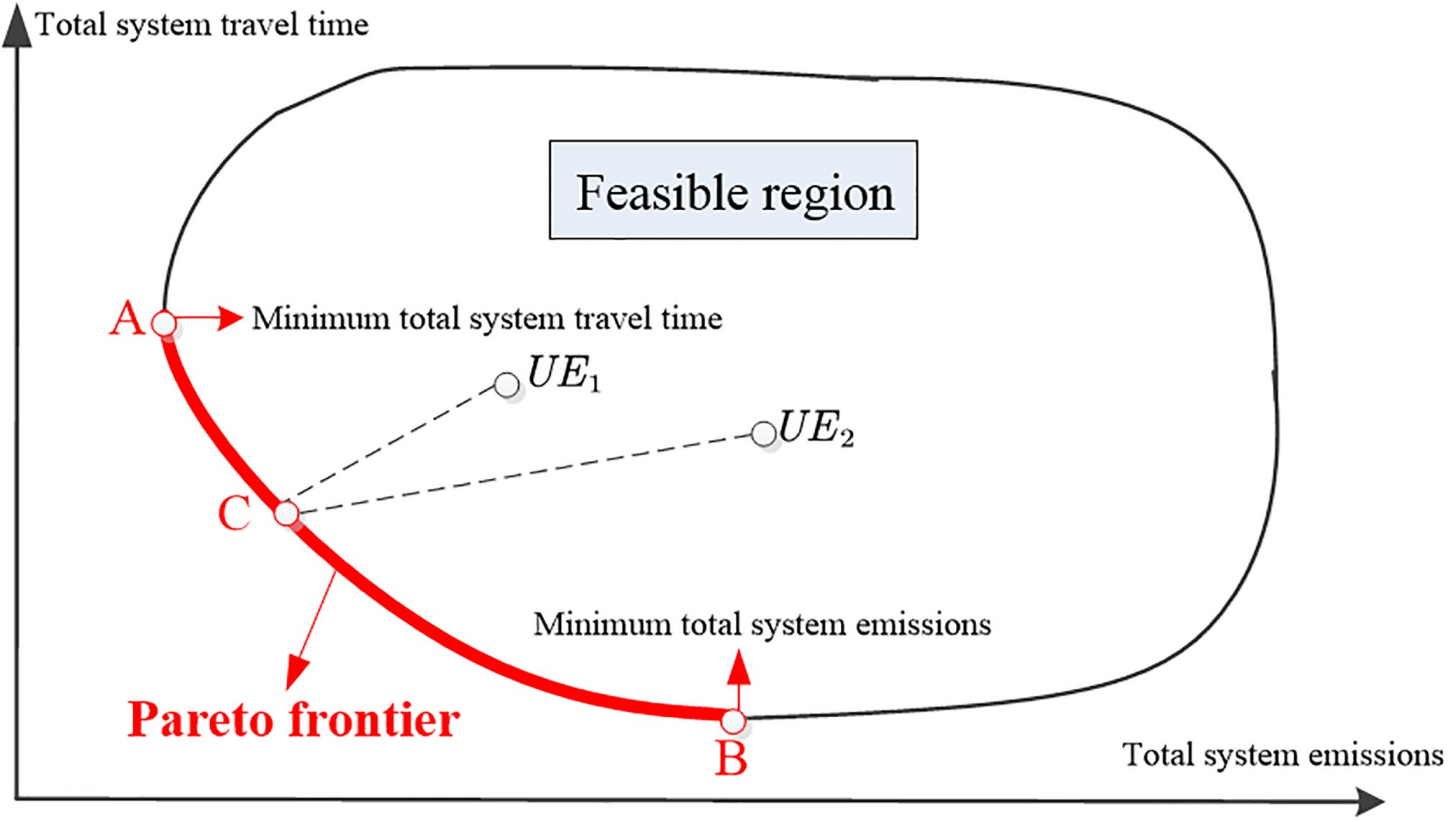
Pareto-efficient flow pattern and user equilibrium flow pattern. The red line represents the Pareto frontier, A and B are Pareto ends representing the minimum total system travel time and minimum total system emissions, respectively.

Here we use Fig 1 to explain the organization of this paper: Section 2 will provide the notations and preliminaries of this research. Section 3 will investigate whether the differentiable road pricing for EVs and GVs can decentralize a pareto-efficient flow pattern as user equilibrium. To be more specific, after implementing differentiable road pricing, travelers still select the route based on Wardrop principle, but pareto-efficient pattern can be achieved from the perspective of system manager. In Fig 1, any user equilibrium flow pattern such as UE_1_ and UE_2_ can be induced into a pareto-efficient pattern (point C). Section 3 will derive the deviation gap of a point on the pareto frontier and explore the following questions: when the point C reach minimum total system travel time (Point A), how far could the corresponding total system emissions deviate from its minimum value? Conversely, when total system emissions are minimized (Point B), how far could the corresponding total system travel time deviate from its minimum value? Section 5 will qualify the efficiency gain of differentiable road pricing by comparing the total system travel time/ total system emissions between the state without tolls and the state with differentiable tolls based on the concept of price of anarchy (POA). In Fig 1, since differentiable road pricing can induce point *UE*_1_ and point *UE*_2_ to point C located on pareto-efficient frontier, we will bound the ratio of total system travel time/ total system emissions at point *UE*_1_ to total system travel time/ total system emissions at point C by defining POA. After obtaining the theoretical bound of POA, the case studies based on two cities will be conducted to calculate the numerical bound of POA and validate the effectiveness of general bound. Section 6 will conclude with a summary of the main findings and suggestions for future studies.

## 2. Notations and preliminaries

### 2.1 Notations

Table 1 summarizes the parameters, variables and abbreviations used in this paper to describe the mathematical models related to the differentiable road pricing scheme.

**Table 1:** Parameters, variables and abbreviations.

EV	Electric vehicles	$c_{a}^{E}$	EV user’s link travel cost
GV	Gasoline vehicles	$c_{a}^{G}$	GV user’s link travel cost
$\tau_{a}^{G}$	Link-based differentiable tolls for GV	*T*	Total system travel time
$\tau_{a}^{E}$	Link-based differentiable tolls for EV	*E*	Total system emissions
$v_{a}^{E}$	Link flow of EV	*η*_*max*_	Upper bound of emission factor
$v_{a}^{G}$	Link flow of GV	*η*_*min*_	Lower bound of emission factor
*v*_*a*_	Aggravated link flow of GV and GV	*$\alpha_{T}^{*}$*	Upper bound of deviation factor *α*_*T*_
$f_{\left(r,w\right)}^{E}$	Path flow of EV on path *r* between OD pair *w*	$\alpha_{E}^{*}$	Upper bound of deviation factor *α*_*E*_
$f_{\left(r,w\right)}^{G}$	Path flow of GV on path *r* between OD pair *w*	*ϕ*	Weighting factor of travel demand level
$d_{w}^{E}$	Travel demand of EV between OD pair *w*	*POA*_*T*_	Price of anarchy for total system travel time
$d_{w}^{G}$	Travel demand of GV between OD pair *w*	*POA*_*E*_	Price of anarchy for total system emissions
*$c_{rw}^{E}$*	EV user’s travel cost on path r between OD pair *w*	*P*	Percentage of electric vehicles
*$c_{rw}^{G}$*	GV user’s travel cost on path r between OD pair *w*	*κ*	Value of time

### 2.2 Preliminaries and definitions

In this paper, a mixed urban network G = (N, A) consisting of pure electric vehicles (EVs) without emissions and gasoline vehicles (GVs) is considered, where N and A represent the set of nodes and links, respectively. We assume that the travel demand *d*_*w*_ for each OD pair *w* ∈ *W* is fixed, and the percentage of EVs is *P*. The set of feasible path flow patterns ($\Omega_{f}^{E},\Omega_{f}^{G}$) and link flow patterns ($\Omega_{v}^{E},\Omega_{v}^{G}$) for the EVs and GVs are expressed in Eqs (1)–(4):

$$\Omega_{f}^{E}=\left\{f^{E}={\left(f_{rw}^{E},r\in R_{w},w\in W\right)}^{T}|\sum_{r\in R_{w}}f_{rw}^{E}=d_{w}^{E},f_{rw}^{E}\geq 0,r\in R_{w},w\in W\right\}$$ (1)

$$\Omega_{f}^{G}=\left\{f^{G}={\left(f_{rw}^{G},r\in R_{w},w\in W\right)}^{T}|\sum_{r\in R_{w}}f_{rw}^{G}=d_{w}^{G},f_{rw}^{G}\geq 0,r\in R_{w},w\in W\right\}$$ (2)

$$\Omega_{v}^{E}=\left\{v^{E}={\left(v_{a}^{E},a\in A\right)}^{T}|v_{a}^{E}=\sum_{r\in R_{w}}\sum_{w\in W}f_{rw}^{E}\delta_{ar}^{w,E},f\in \Omega_{f}^{E},a\in A\right\}$$ (3)

$$\Omega_{v}^{G}=\left\{v^{G}={\left(v_{a}^{G},a\in A\right)}^{T}|v_{a}^{G}=\sum_{r\in R_{w}}\sum_{w\in W}f_{rw}^{G}\delta_{ar}^{w,G},f\in \Omega_{f}^{G},a\in A\right\}$$ (4)

where $\delta_{ar}^{w,E}/\delta_{ar}^{w,G}$ indicates (0 or1) whether route *r* (*r* ∈ *R*_*w*_) chosen by EV/GV users passes through link *a* (*a* ∈ *A*). Moreover, the relationship between EV and GV users’ path travel cost and link travel cost can be expressed as shown in Eqs (5) and (6):

$$c_{rw}^{E}=\sum_{a\in A}c_{a}^{E}\left(v_{a}^{E}\right)\delta_{ar}^{w,E},a\in A,r\in R_{w},w\in W$$ (5)

$$c_{rw}^{G}=\sum_{a\in A}c_{a}^{G}\left(v_{a}^{G}\right)\delta_{ar}^{w,G},a\in A,r\in R_{w},w\in W$$ (6)

When there is no road pricing scheme, a traveler’s link travel cost is $c_{a}^{E}(v)=\kappa t_{a}(v)$. Under differentiable tolls $\tau =(\tau_{a}^{G},\tau_{a}^{E},a\in A)$, an EV user’s cost is $c_{a}^{E}(v)=\kappa t_{a}(v)+\tau_{a}^{E}$, and a GV user’s cost is $c_{a}^{G}(v)=\kappa t_{a}(v)+\tau_{a}^{G}$. This research investigates whether path flow vector $f^{*}=(f_{rw}^{E*},f_{rw}^{G*})$ and minimum OD cost vector $\mu =(\mu_{w}^{E},\mu_{w}^{G})$ exists, so that for each *r* ∈ *R*_*w*_, *w* ∈ *W*, the mixed equilibrium conditions for EVs and GVs hold (Eqs (7) and (8)).

$$c_{rw}^{E}-\mu_{w}^{E}\{\begin{array}{c} \begin{array}{ccc} =0 & if & f_{rw}^{E^{*}}>0 \end{array}\\ \begin{array}{ccc} \geq 0 & if & f_{rw}^{E^{*}}=0 \end{array} \end{array}$$ (7)

$$c_{rw}^{G}-\mu_{w}^{G}\{\begin{array}{c} \begin{array}{ccc} =0 & if & f_{rw}^{G^{*}}>0 \end{array}\\ \begin{array}{ccc} \geq 0 & if & f_{rw}^{G^{*}}=0 \end{array} \end{array}$$ (8)

where $\mu_{w}^{E}$ and $\mu_{w}^{G}$ represent the EV and GV users’ minimum path travel costs for OD pair *w*, *w* ∈ *W*. The BPR (US Bureau of Public Roads) link time function is used in this paper and assumes that the value of time for EV and GV users (*κ*) is the same.

Assuming that the value of time for EV and GV users is the same, this paper uses the BPR (US Bureau of Public Roads) link time function (9):

$$t_{a}\left(v_{a}\right)=t_{0}\left(1+\alpha {\left(\frac{v_{a}}{C_{a}}\right)}^{\beta }\right)$$ (9)

Since more than 90% [32] of the carbon monoxide (CO) in urban centers is from vehicular emissions, Wallace et al. [33] used a nonlinear macroscopic model to estimate the emission load of link-based vehicular CO, and the coefficients are equivalent to those in TRANSYT-7F. The emission functions of such a form (10) are widely used; for example, they are used in [11,34–36].

$$e_{a}\left(v_{a}\right)=ROP_{a}\cdot l_{a}=0.2038t_{a}\left(v_{a}\right)\cdot \text{exp}\left(0.7962\left(l_{a}/t_{a}\left(v_{a}\right)\right)\right)$$ (10)

As long as *t*_*a*_(*v*_*a*_) is strictly increasing and the speed *L*_*a*_/(*t*_*a*_(*v*_*a*_)) < 1/0.7962 = 75 km/h, *d*^2^*t*_*a*_(*v*_*a*_)/*d*(*v*_*a*_)^2^ > 0, then, *d*^2^*e*_*a*_(*v*_*a*_)/*d*(*v*_*a*_)^2^> 0. In other words, the total CO emission function *e*_*a*_(*v*_*a*_). *v*_*a*_ is strictly convex. The relationship between *e*_*a*_(*v*_*a*_) and *t*_*a*_(*v*_*a*_) is given in Eq (11):

$$e_{a}\left(v_{a}\right)=\eta_{a}\left(v_{a}\right)\cdot t_{a}\left(v_{a}\right)$$ (11)

where *ROP*_*a*_ · *l*_*a*_ = 0.2038 · exp(0.7962(*l*_*a*_/*t*_*a*_(*v*_*a*_))), *l*_*a*_ denotes the length (km) of a link and *t*_*a*_(*v*_*a*_) denotes the link travel time. If the maximum speed is 75 (km/h), then *η*(*v*_*a*_) ≤ 0.5513, and thus, *η*_*min*_ can be obtained. If the minimum speed is set to 0 (km/h), then we have η(v_a_) ≥ 0.2038.

Due to the development of clean energy technology, this paper focuses only on emissions in urban traffic networks and ignores emissions from the power grid. Although both EVs and GVs contribute to the total system travel time, since only GVs contribute to the total system emissions, a bi-objective optimization program with the objective function (12) can be obtained:

$$\underset{v^{G}\in \Omega_{v}^{G},v^{E}\in \Omega_{v}^{E}}{\text{min}}\left(\begin{array}{c} T\left(v\right)\\ E\left(v\right) \end{array}\right)=\left(\begin{array}{c} \sum_{a\in A}t_{a}\left(v_{a}^{G}+v_{a}^{E}\right)\cdot \left(v_{a}^{G}+v_{a}^{E}\right)\\ \sum_{a\in A}e_{a}\left(v_{a}^{G}+v_{a}^{E}\right)\cdot v_{a}^{G} \end{array}\right)$$ (12)

Let $v^{*}\in \Omega_{v}^{*}$ denote the set of Pareto-efficient solutions. The Pareto-efficient link flow pattern is defined as follows:

**Definition 1.** Pareto-efficient flow pattern

When a link flow pattern $v^{*}\in \Omega_{v}^{*}$ is Pareto-efficient optimal, neither the total system travel time *T* (v*) nor the total system emissions *E* (v*) can be reduced without increasing the other. Specifically, there is no other pattern $v^{*}\in \Omega_{v}^{*}$ that satisfies *E*(**v**) ≤ *E*(v*) and *T*(**v**) ≤ *T*(v*), and at least one of the inequalities holds: *E*(**v**) < *E*(v*), *T*(**v**) < *T*(v*).

Basically, we calculate the emissions and travel time based on volume, and the objective is to minimize the total cost of both based on weighting factors for each. Since both *T*(**v**) and *E*(**v**) are convex, their Hessian matrices are both positive definite, and thus, the Pareto-efficient solution can be obtained by solving the weighting bi-objective mathematical problem (13):

$$\begin{array}{cc} \text{min} & \omega_{1}\sum_{a\in A}t_{a}\left(v_{a}^{E}+v_{a}^{G}\right)\cdot \end{array}\left(v_{a}^{E}+v_{a}^{G}\right)+\omega_{2}\sum_{a\in A}e_{a}\left(v_{a}^{E}+v_{a}^{G}\right)\cdot v_{a}^{G}$$ (13)

subject to constraints (1)-(4).

From the KKT conditions of the bi-objective optimization program (1)-(4), (13), we can conclude that for any optimal solution, the ($f_{e}^{*},v_{e}^{*}$) and ($f_{g}^{*},v_{g}^{*}$), conditions (14)-(15) are always satisfied.

$$\begin{array}{cc} c_{rw}^{E}=\mu_{w}^{E} & if \end{array}\begin{array}{cc} f_{rw}^{E^{*}}>0; & c_{rw}^{E}\geq \mu_{w}^{E} \end{array}\begin{array}{cc} if & f_{rw}^{E^{*}}=0 \end{array}$$ (14)

$$\begin{array}{cc} c_{rw}^{G}=\mu_{w}^{G} & if \end{array}\begin{array}{cc} f_{rw}^{G^{*}}>0; & c_{rw}^{G}\geq \mu_{w}^{G} \end{array}\begin{array}{cc} if & f_{rw}^{G^{*}}=0 \end{array}$$ (15)

where $\mu_{w}^{E}$ and $\mu_{w}^{G}$ are Lagrange multipliers corresponding to constraints (1) and (2), respectively. It is evident that conditions (14)-(15) are consistent with the user equilibrium conditions of the EVs and GV users (7) and (8).

**Definition 2.** Differentiable marginal road pricing

This research proposes a differentiable link-based marginal road pricing, differentiable road pricing or differentiable tolls for short, to manage congestion and emissions simultaneously in the urban networks with EVs and GVs. We investigate whether differentiable marginal pricing for EVs and GVs can induce the user equilibrium flow pattern into pareto optimum flow pattern by considering the congestion and emissions simultaneously. The optimal toll on a link is equal to the difference between the marginal social cost and the marginal private cost, which can internalize the user externalities including congestion part and emissions part, and thus achieve a Pareto-optimal efficient pattern in the network with EVs and GVs.

From the KKT conditions of bi-objective optimization program (1)-(4), (13), we can conclude that for any optimal solutions ($f_{e}^{*},v_{e}^{*}$) and ($f_{g}^{*},v_{g}^{*}$), conditions (14)-(15) always satisfy.

$$\begin{array}{cc} c_{rw}^{E}=\mu_{w}^{E} & if \end{array}\begin{array}{cc} f_{rw}^{E^{*}}>0; & c_{rw}^{E}\geq \mu_{w}^{E} \end{array}\begin{array}{cc} if & f_{rw}^{E^{*}}=0 \end{array}$$ (16)

$$\begin{array}{cc} c_{rw}^{G}=\mu_{w}^{G} & if \end{array}\begin{array}{cc} f_{rw}^{G^{*}}>0; & c_{rw}^{G}\geq \mu_{w}^{G} \end{array}\begin{array}{cc} if & f_{rw}^{G^{*}}=0 \end{array}$$ (17)

where $\mu_{w}^{E}$ and $\mu_{w}^{G}$ are Lagrange multipliers correspond to constraints (1) and (2), respectively. It is evident that conditions (14)-(15) are consistent with user equilibrium condition of EVs and GV users (7) and (8). According to the first order condition of the bi-objective program (1)-(4), (13), the differentiable road pricing for EV and GV can be expressed as (18) and (19):

$$\tau_{a}^{E}=\omega_{1}\sum_{a\in A}\left(v_{a}\right)\cdot \left(\frac{\partial t_{a}\left(v_{a}\right)}{\partial v_{a}}\right)\delta_{ar}^{w,E}+\omega_{2}\left(v_{a}^{G}\cdot \frac{\partial e_{a}\left(v_{a}\right)}{\partial v_{a}}\right)\delta_{ar}^{w,E}$$ (18)

$$\tau_{a}^{G}=\omega_{1}\sum_{a\in A}\left(v_{a}\right)\cdot \left(\frac{\partial t_{a}\left(v_{a}\right)}{\partial v_{a}}\right)\delta_{ar}^{w,G}+\omega_{2}\sum_{a\in A}\left(e_{a}\left(v_{a}\right)+v_{a}^{G}\frac{\partial e_{a}\left(v_{a}\right)}{\partial v_{a}^{G}}\right)\delta_{ar}^{w,G}$$ (19)

## 3. Decentralization of differentiable tolls

In this section, we investigate whether the differentiable road pricing can induce user equilibrium into two-criteria pareto optimal in terms of congestion and emissions, and explore whether the differentiable can decentralize a Pareto-efficient flow pattern as a unique user equilibrium flow pattern.

**Proposition 1.** The Pareto-efficient flow pattern $v^{G*}\in \Omega_{v}^{G*},v^{E*}\in \Omega_{v}^{E*}$ *can be decentralized as user equilibrium by nonnegative differentiable tolls*.

**Proof.** Since Pareto-efficient solutions can be obtained by solving the bi-objective problem (12) we let $\Omega_{v}^{*}=(\Omega_{v}^{E*},\Omega_{v}^{G*})$ denote the set of Pareto-efficient solutions to the problem and investigate whether any Pareto-efficient link flow pattern $v^{*}=(v^{E*},v^{G*})(v^{E*}\epsilon \Omega_{v}^{E*},v^{G*}\in \Omega_{v}^{G*})$ can be decentralized as a user equilibrium pattern by differentiable tolls.

First, consider the following linear programming (LP) problem:

$$\underset{f}{\text{min}}\sum_{a\in A}t_{a}\left(v^{*}\right)\cdot \left(v_{a}^{E}+v_{a}^{G}\right)$$ (20)

subject to

$$\sum_{w\in W}\sum_{r\in R_{w}}\sum_{m\in M}f_{rw}^{E}\delta_{ar}^{w}\leq v_{a}^{*},a\in A$$ (21)

$$\sum_{w\in W}\sum_{r\in R_{w}}\sum_{m\in M}f_{rw}^{G}\delta_{ar}^{w}\leq v_{a}^{G*},a\in A$$ (22)

$$\sum_{r\in R_{w}}f_{rw}^{E}-d_{w}^{E}=0,w\in W,r\in R_{w}$$ (23)

$$\sum_{r\in R_{w}}f_{rw}^{G}-d_{w}^{G}=0,w\in W,r\in R_{w}$$ (24)

$$f_{rw}^{E},f_{rw}^{G}\geq 0,\;r\in R_{w},w\in W$$ (25)

The dual problem of programming (26)-(29) is shown as follows:

$$\underset{\lambda ,\mu }{\text{max}}\;\sum_{a\in A}v_{a}^{E*}\lambda_{a}^{E}+v_{a}^{G*}\lambda_{a}^{G}+\sum_{w\in W}d_{w}^{E}\mu_{w}^{E}+d_{w}^{G}\mu_{w}^{G}$$ (26)

subject to

$$\lambda_{a}^{E}\delta_{ar}^{w,E}+\mu_{w}^{E}\leq \sum_{a\in A}\kappa t_{a}\left(v^{*}\right)\delta_{ar}^{w,E},a\in A,w\in W,r\in R_{w}$$ (27)

$$\lambda_{a}^{G}\delta_{ar}^{w,G}+\mu_{w}^{G}\leq \sum_{a\in A}\kappa t_{a}\left(v^{*}\right)\delta_{ar}^{w,G},a\in A,w\in W,r\in R_{w}$$ (28)

$$\lambda_{a}^{G},\lambda_{a}^{G}\leq \text{0}$$ (29)

where $\lambda_{a}^{G}$ and $\lambda_{a}^{E}$, a ∈ A are Lagrange multipliers related to constraints (21) and (22) and $\mu_{w}^{G}(w\in W)$ and $\mu_{w}^{E}(w\in W)$ are vectors of multipliers associated with the constraints (23) and (24). Let $\tau_{a}^{E}=-\lambda_{a}^{E}$, $\tau_{a}^{G}=-\lambda_{a}^{G}$. Then, inequalities (27) and (28) can be rewritten as inequalities (30)-(31):

$$\sum_{a\in A}\left\{\left[t_{a}\left(v^{*}\right)+\tau_{a}^{E}\right]\delta_{ar}^{w,E}-\mu_{w}^{E}\right\}\geq 0,w\in W,r\in R_{w}$$ (30)

$$\sum_{a\in A}\left\{\left[t_{a}\left(v^{*}\right)+\tau_{a}^{G}\right]\delta_{ar}^{w,G}-\mu_{w}^{G}\right\}\geq 0,w\in W,r\in R_{w}$$ (31)

At the optimal points of the primal and dual problems, the following complementary slackness conditions will satisfy:

$$\left\{\sum_{a\in A}\left[\kappa t_{a}\left(v^{*}\right)+\tau_{a}^{E}\right]\delta_{ar}^{w,E}-\mu_{w}^{E}\right\}f_{rw}^{E}=0,w\in W,r\in R_{w}$$ (32)

$$\left\{\sum_{a\in A}\left[\kappa t_{a}\left(v^{*}\right)+\tau_{a}^{G}\right]\delta_{ar}^{w,G}-\mu_{w}^{G}\right\}f_{rw}^{G}=0,w\in W,r\in R_{w}$$ (33)

Eqs (32) and (33) are alternatives of the user equilibrium conditions (7)-(8). Since we have proven that optimal solutions of the LP problem can be decentralized as a UE flow pattern by differentiable tolls, we investigate whether v*is the optimal solution of the LP problem through proof by contradiction.

Assume that v* is not the optimal solution of the LP programming (26)-(29), and **v**′ is the optimal solution instead: v* ≠ **v**′. According to the definition of Pareto-efficient flows, we can obtain $v_{a}^{E\prime }\leq v_{a}^{E*}$ or $v_{a}^{G\prime }\leq v_{a}^{G*}$.

Since *t*_*a*_(·) is increasing with any link flow: *t*_*a*_(**v**′) ≤ *t*_*a*_(v*), *a* ∈ *A*. Therefore,

$$t_{a}\left(v^{\prime }\right)v_{a}^{E\prime }<t_{a}\left(v^{*}\right)v_{a}^{E*}$$ (34)

$$t_{a}\left(v^{\prime }\right)v_{a}^{G\prime }<t_{a}\left(v^{*}\right)v_{a}^{G*}$$ (35)

$$t_{a}\left(v^{\prime }\right)\left(v_{a}^{E\prime }+v_{a}^{G\prime }\right)<t_{a}\left(v^{*}\right)\left(v_{a}^{E*}+v_{a}^{G*}\right)$$ (36)

Similarly, *e*_*a*_(·) increases with any link flow pattern: *e*_*a*_(**v**′) ≤ *e*_*a*_(v*), *a* ∈ *A*.

$$e_{a}\left(v^{\prime }\right)v_{a}^{G\prime }<e_{a}\left(v^{*}\right)v_{a}^{G*}$$ (37)

Since inequalities (36) and (37) conflict with the definition of Pareto-efficient link flows (Definition 1), the assumption is false, and **v**′ is not the optimal solution, implying that v* can be decentralized as user equilibrium. Thus, Proposition 1 can be concluded.

Since this bi-objective optimization problem involves with different vehicle types, and thus the link flow interactions among them cannot be ignored. Considering the interactions among link emission function *e*_*a*_(·) and link travel time function *t*_*a*_(·), the Jacobian of vector *t*_*a*_(**v**) is a matrix ∇_v_*t*(**v**) with the dimension of (2*n*) × (2*n*), and the Jacobian of vector *e*_*a*_(**v**) is a matrix ∇_v_*e*(**v**) with the dimension of *n* × 2*n*. Since only adjacent links have flow interactions, ∇_v_*t*(**v**) and ∇_v_*e*(**v**) are sparse matrices in most cases (Appendix 1).

Since the link flow interactions are asymmetric, this two-class mixed equilibrium link flow pattern cannot be obtained by the solution of an equivalent mathematical program but can be formulated as a variation inequality (VI) problem (38):

$$\sum_{a\in A}\left[c_{a}^{E}\left(\bar{v}\right)\right]\left(v_{a}^{E}-{\bar{v}}_{a}^{E}\right)+\sum_{a\in A}\left[c_{a}^{G}\left(\bar{v}\right)\right]\left(v_{a}^{G}-{\bar{v}}_{a}^{G}\right)\geq 0$$ (38)

**Proof.** First, we prove that

$$\left[c_{rw}^{E}\left(\bar{f}\right)-\mu_{w}^{E}\right]\left(f_{rw}^{E}-{\bar{f}}_{rw}^{E}\right)\geq 0,w\in W,r\in R_{w}$$ (39)

$$\left[c_{rw}^{G}\left(\bar{f}\right)-\mu_{w}^{G}\right]\left(f_{rw}^{G}-{\bar{f}}_{rw}^{G}\right)\geq 0,w\in W,r\in R_{w}$$ (40)

Taking EV as an example, considering the user equilibrium condition (14), if ${\overset{-}{f}}_{rw}^{E}\geq 0$, then $c_{rw}^{E}(\overset{-}{f})-\mu_{w}^{E}=0$. If ${\overset{-}{f}}_{rw}^{E}=0$, then $c_{rw}^{E}(\overset{-}{f})-\mu_{w}^{E}\geq 0$. Thus, for any ${\overset{-}{f}}_{rw}^{E}\geq 0$, inequality (39) holds. For GVs, inequality (40) holds. By summing inequality (39) and inequality (39) and summarizing the inequalities over all paths, *r* ∈ *R*_*w*_, the OD pairs ω ∈ W, and we obtain inequality (41):

$$\sum_{w\in W}\sum_{r\in R_{w}}\left[c_{rw}^{E}\left(\bar{f}\right)-\mu_{w}^{E}\right]\left(f_{rw}^{E}-{\bar{f}}_{rw}^{E}\right)+\sum_{w\in W}\sum_{r\in R_{w}}\left[c_{rw}^{G}\left(\bar{f}\right)-\mu_{w}^{G}\right]\left(f_{rw}^{G}-{\bar{f}}_{rw}^{G}\right)\geq 0$$ (41)

$$\begin{array}{l} \sum_{w\in W}\sum_{r\in R_{w}}\left[c_{rw}^{E}\left(\bar{f}\right)\right]\left(f_{rw}^{E}-{\bar{f}}_{rw}^{E}\right)+\sum_{w\in W}\sum_{r\in R_{w}}\left[c_{rw}^{G}\left(\bar{f}\right)\right]\left(f_{rw}^{G}-{\bar{f}}_{rw}^{G}\right)\\ \geq \sum_{w\in W}\sum_{r\in R_{w}}\left[\mu_{w}^{E}\left(f_{rw}^{E}-{\bar{f}}_{rw}^{E}\right)+\mu_{w}^{G}\left(f_{rw}^{G}-{\bar{f}}_{rw}^{G}\right)\right] \end{array}$$ (42)

The right-hand side (RHS) of inequality (42) can be rewritten as:

$$\sum_{w\in W}\left[\mu_{w}^{E}\left(d_{w}^{E}-d_{w}^{E}\right)+\mu_{w}^{G}\left(d_{w}^{G}-d_{w}^{G}\right)\right]=0$$ (43)

The left-hand side (LHS) of inequality(42) can be rewritten as (44):

$$\begin{array}{l} \sum_{w\in W}\sum_{r\in R_{w}}\left[c_{rw}^{E}\left(\bar{f}\right)\right]\left(f_{rw}^{E}-{\bar{f}}_{rw}^{E}\right)+\sum_{w\in W}\sum_{r\in R_{w}}\left[c_{rw}^{G}\left(\bar{f}\right)\right]\left(f_{rw}^{G}-{\bar{f}}_{rw}^{G}\right)\\ =\sum_{w\in W}\sum_{r\in R_{w}}\left[c_{a}^{E}\left(\bar{v}\right)\right]\left(v_{a}^{E}-{\bar{v}}_{a}^{E}\right)+\sum_{w\in W}\sum_{r\in R_{w}}\left[c_{a}^{G}\left(\bar{v}\right)\right]\left(v_{a}^{G}-{\bar{v}}_{a}^{G}\right) \end{array}$$ (44)

We prove that the solution of variation inequality (VI) (38) is a UE flow pattern. From inequality (38) and (42), we have:

$$\text{min}\sum_{w\in W}\sum_{r\in R_{w}}\left\{\left[c_{rw}^{E}\left(\bar{f}\right)\right]\cdot f_{rw}^{E}+\left[c_{rw}^{G}\left(\bar{f}\right)\right]\cdot f_{rw}^{G}\right\}\geq \sum_{w\in W}\sum_{r\in R_{w}}\left\{\left[c_{rw}^{E}\left(\bar{f}\right)\right]{\bar{f}}_{rw}^{E}+\left[c_{rw}^{G}\left(\bar{f}\right)\right]{\bar{f}}_{rw}^{G}\right\}$$ (45)

where the right-hand side (RHS) of inequality (45) is a constant and the left-hand side (LHS) is a linear program. Inequality (45) shows that $\bar{f}$ is the optimal solution of the linear program, and the first-order conditions of the minimization program is (23)-(25) and (46)-(49).

$$f_{rw}^{G*}\left[c_{rw}^{G}\left(\bar{f}\right)-\mu_{w}^{G}\right]=0,r\in R_{w},w\in w$$ (46)

$$c_{rw}^{G}\left(\bar{f}\right)-\mu_{w}^{G}\geq 0,r\in R_{w},w\in w$$ (47)

$${\bar{f}}_{rw}^{E}\left[c_{rw}^{E}\left(\bar{f}\right)-\mu_{w}^{E}\right]=0,r\in R_{w},w\in w$$ (48)

$$c_{rw}^{E}\left(\bar{f}\right)-\mu_{w}^{E}\geq 0,r\in R_{w},w\in w$$ (49)

Taking GV users as an example, if ${\overset{-}{f}}_{rw}^{G}>0$, then $c_{rw}^{G}(f)-\mu_{w}^{G}=0$. If ${\overset{-}{f}}_{rw}^{G}=0$, then $c_{rw}^{G}(f)-\mu_{w}^{G}\geq 0$ for EV users, and thus, it can be concluded that **f** is the UE flow pattern. Since both $c_{rw}^{G}$ and $c_{rw}^{E}$ are continuous and the solution sets in (38) are compact, the solution to variation inequality (38) always exists.

To analyze the stability and validity of the equilibrium, Proposition 3 is given to explore the uniqueness of the equilibrium for the two-criteria problem.

**Proposition 2.** *Differentiable road pricing for E*Vs *and G*Vs *can decentralize the unique vehicle type-specific link flow pattern*.

**Proof.** Let **v**^1^ and **v**^2^ denote any two types of aggregate link flow patterns. Assuming that **v**^1^ and **v**^2^ are in user equilibrium, according to variation inequality (38), we have:

$$\sum_{a\in A}\left[c_{a}^{E}\left(v^{1}\right)\right]\left(v_{a}^{E2}-v_{a}^{E1}\right)+\sum_{a\in A}\left[c_{a}^{G}\left(v^{1}\right)\right]\left(v_{a}^{G2}-v_{a}^{G1}\right)\geq 0$$ (50)

$$\sum_{a\in A}\left[c_{a}^{E}\left(v^{2}\right)\right]\left(v_{a}^{E1}-v_{a}^{E2}\right)+\sum_{a\in A}\left[c_{a}^{G}\left(v^{2}\right)\right]\left(v_{a}^{G1}-v_{a}^{G2}\right)\geq 0$$ (51)

After negating inequalities (HYPERLINK 50) and (51) summarizing them, we obtain:

$$\sum_{a\in A}\left[c_{a}^{E}\left(v^{1}\right)-c_{a}^{E}\left(v^{2}\right)\right]\cdot \left(v_{a}^{E1}-v_{a}^{E2}\right)+\sum_{a\in A}\left[c_{a}^{G}\left(v^{1}\right)-c_{a}^{G}\left(v^{2}\right)\right]\cdot \left(v_{a}^{G1}-v_{a}^{G2}\right)\leq 0$$ (52)

Since the link cost function of EVs and GVs is strictly increasing, we obtain:

$$\sum_{a\in A}\left[c_{a}^{E}\left(v^{1}\right)-c_{a}^{E}\left(v^{2}\right)\right]\cdot \left(v_{a}^{E1}-v_{a}^{E2}\right)+\sum_{a\in A}\left[c_{a}^{G}\left(v^{1}\right)-c_{a}^{G}\left(v^{2}\right)\right]\cdot \left(v_{a}^{G1}-v_{a}^{G2}\right)\geq 0$$ (53)

From inequality (52) and inequality (53), we obtain:

$$\sum_{a\in A}\left[c_{a}^{E}\left(v^{1}\right)-c_{a}^{E}\left(v^{2}\right)\right]\cdot \left(v_{a}^{E1}-v_{a}^{E2}\right)+\sum_{a\in A}\left[c_{a}^{G}\left(v^{1}\right)-c_{a}^{G}\left(v^{2}\right)\right]\cdot \left(v_{a}^{G1}-v_{a}^{G2}\right)=0$$ (54)

Since **v**^1^ and **v**^2^ denote any two types of aggregate link flow patterns, we have $c_{a}^{E}\left(v^{1}\right)\neq c_{a}^{E}\left(v^{2}\right)$ and $c_{a}^{G}\left(v^{1}\right)\neq c_{a}^{G}\left(v^{2}\right)$. According to Eq (54), we obtain $v_{a}^{E1}=v_{a}^{E2}$ and $v_{a}^{G1}=v_{a}^{G2}$, thus Proposition 2 can be concluded.

## 4. Deviation gap between the two-criteria system optima

### 4.1 Bound of the deviation factors

The solution set of problem (12) naturally forms a Pareto frontier (Fig 1), in which each point represents a Pareto optimum. Considering this two-criteria system under differentiable road pricing, we explore the gap deviation between the total system travel time (*T*) and total system emissions (*E*) in the Pareto optimum and their respective single-criterion-based system optimum.

By changing the percentage of EVs, *T*(**v**) remains constant, while *E*(**v**) varies within a range; this range can be bounded via Lemma 1:

**Lemma 1.** *For any feasible link flow pattern* **v** *under differentiable tolls*, *total system emissions E*(**v**) *can be bounded by total system travel time T* (**v**):

$$\left(1-P\right)\eta_{\text{min}}T\left(v\right)\leq E\left(v\right)\leq \frac{\left(\eta_{\text{max}}+1-P\right)}{\left(1+1/\eta_{\text{max}}\right)}T\left(v\right)$$ (55)

**Proof.** See in Appendix 2.

**Proposition 3.** *It holds that*

$$1\leq \alpha_{T}^{*}\alpha_{E}^{*}\leq \frac{\eta_{\text{max}}+1-P}{\eta_{\text{min}}\left(1-P\right)\left(1+1/\eta_{\text{max}}\right)}$$ (56)

**Proof.** According to the definition of deviation factors, we have:

$$\alpha_{T}^{*}\left(v\right)\cdot \alpha_{E}^{*}\left(v\right)=\frac{T\left(v^{ME}\right)}{E\left(v^{ME}\right)}\cdot \frac{E\left(v^{SO}\right)}{T\left(v^{SO}\right)}$$ (57)

By substituting **v**^*ME*^ and **v**^*So*^ into Lemma 1, we have:

$$\frac{T\left(v^{ME}\right)}{E\left(v^{ME}\right)}\leq \frac{\text{1}}{\eta_{\text{min}}\left(1-P\right)}$$ (58)

$$\frac{E\left(v^{SO}\right)}{T\left(v^{SO}\right)}\leq \frac{\eta_{\text{max}}+1-P}{\left(1+1/\eta_{\text{max}}\right)}$$ (59)

Thus, Proposition 3 can be concluded.

### 4.2 Numerical example

A numerical example is conducted. Travel demand *d*_*w*_ is set as 1000, the link-based travel time function is *t*_*a*_(*v*_*a*_) = 0.5 +0.15 (*v*_*a*_/*c*_*a*_)^4^, and the link-based emission function is *e*_*a*_(*v*_*a*_) = 0.2038 ·*t*_*a*_(*v*_*a*_) · exp(0.7962 ·(*l*_*a*_/*t*_*a*_(*v*_*a*_))). The percentage of EVs (*P*) are changed from 0.1 to 0.9, and the results for $\alpha_{E}^{*}$ and $\alpha_{T}^{*}$ are calculated. These results are summarized in Table 2.

**Table 2:** Numerical results for the bounds of the deviation factors.

*P*	*T*(v^*ME*^) min	*T*(v^*SO*^) min	*E*(v^*SO*^) g/min	*E*(v^*ME*^) g/min	$\alpha_{T}^{*}$	$\alpha_{T}^{*}$	$\alpha_{T}^{*}\alpha_{E}^{*}$	$\frac{\eta_{\text{max}}+1-P}{\eta_{\text{min}}\left(1-P\right)\left(1+1/\eta_{\text{max}}\right)}$
0.1	265373	215400	59406	41688	1.232	1.425	1.756	2.811
0.2	356272	215400	52789	41596	1.654	1.270	2.101	2.945
0.3	273127	215400	46171	38702	1.268	1.193	1.513	3.116
0.4	459664	215400	39551	34847	2.134	1.135	2.422	3.345
0.5	553363	215400	32940	31544	2.569	1.044	2.682	3.665
0.6	440493	215400	26320	13082	2.045	2.012	4.115	4.146
0.7	438926	215400	19703	9130	2.038	2.158	4.398	4.947
0.8	436896	215400	13091	6342	2.028	2.064	4.186	6.549
0.9	724821	215400	6478	3170	3.365	2.044	6.878	11.354

**Fig 2:**
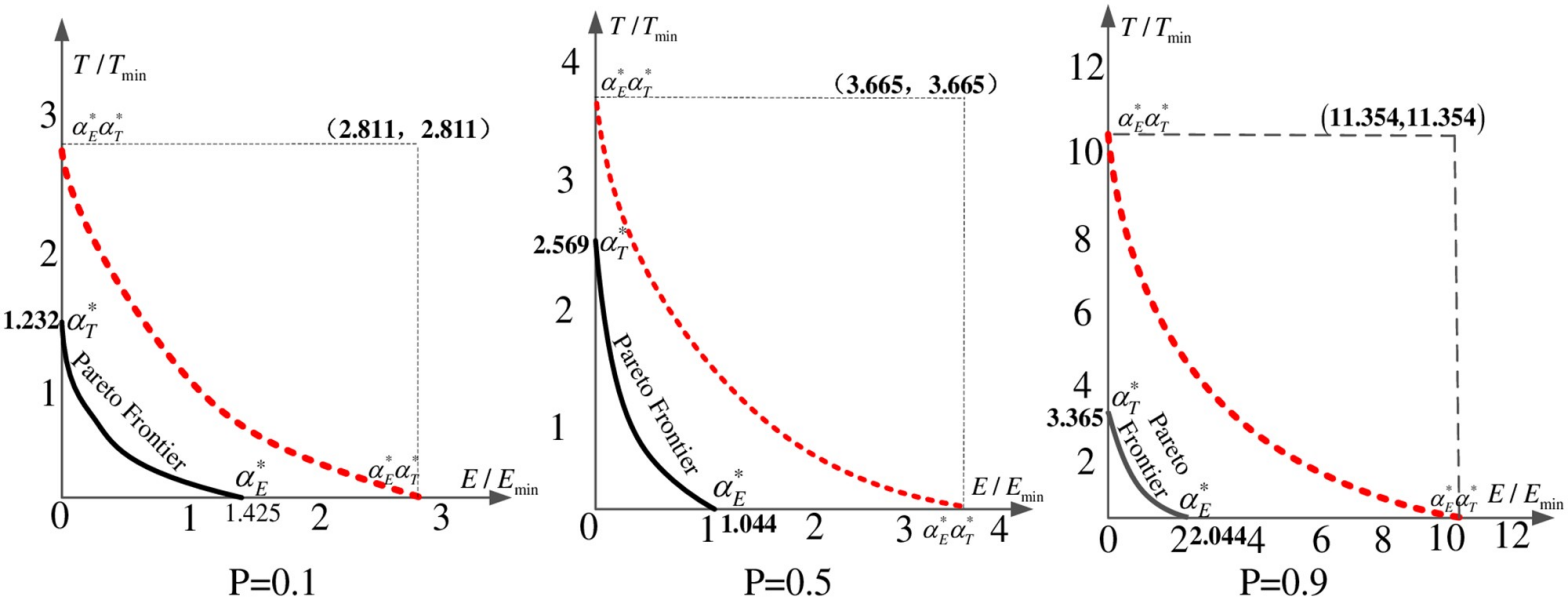
Theoretical and numerical bounds of the deviation factors. The black solid line represents the numerical Pareto frontier, and the red dotted lines represent the theoretical bounds of the deviation factors ${(\alpha }_{E}^{*}\alpha_{T}^{*})$.

After obtaining the numerical results for the deviation factors, we selected three of these factors to plot the results in Fig 2, where the values of *P* are 0.1, 0.5 and 0.9. It is shown that the theoretical results of the deviation factors given in Proposition 3 can always bound the calculated results.

## 5. Bounding the efficiency gain of differentiable road pricing

To qualify how much this two-class two-criteria system will be improved after imposing differentiable road pricing, the efficiency gain of differentiable road pricing will be bounded by the concept of the price of anarchy. Here, the price of anarchy for total system travel time (*POA*_*T*_) and the price of anarchy for total system emissions (*POA*_*E*_) are used to evaluate the performance of differentiable road pricing while managing congestion and emissions, respectively.

### 5.1 Theoretical bound of price of anarchy

**Definition 3.** Price of anarchy for the total system travel time

The price of anarchy for the total system travel time (*POA*_*T*_) is the ratio of the total system travel time in user equilibrium state without tolls to that in pareto-efficient optimal state with differentiable tolls and is defined in Eq (60):

$$POA_{T}=\frac{T(\bar{v})}{T(\hat{v})}=\frac{\langle t({\bar{v}}^{E}+{\bar{v}}^{G}),{\bar{v}}^{E}+{\bar{v}}^{G}\rangle }{\langle t({\hat{v}}^{E}+{\hat{v}}^{G}),{\hat{v}}^{E}+{\hat{v}}^{G}\rangle }$$ (60)

where ${\overset{-}{v}}^{E}$ and ${\overset{-}{v}}^{G}$ represent the link flow patterns of EVs and GVs without tolls, and ${\hat{v}}^{E}$ and ${\hat{v}}^{G}$ represent the link flow patterns of EVs and GVs with differentiable tolls. The upper bound of *POA*_*T*_ is given in Proposition 4.

**Proposition 4.** *Considering the upper bound of POA*_*T*_, *it holds that*:

$$POA_{T}\leq \frac{1}{1-{\left(\beta +1\right)}^{-\frac{1}{\beta }}\cdot \frac{\beta }{\beta +1}}$$ (61)

**Proof.** See in Appendix 3.

The theoretical bound of *POA*_*T*_ is only related to parameter *β* in BPR function (9), it is proved that the upper bound of *POA*_*T*_ is independent on the urban network structure and percentage of electric vehicles.

**Definition 4.** Price of anarchy for the total system emissions

The price of anarchy for total system emissions (*POA*_*E*_) is the ratio of total system emissions in user equilibrium state without tolls to total system emissions in pareto-efficient optimal state with differentiable tolls. It is defined in (62):

$$POA_{E}\text{=}\frac{E(\bar{v})}{E(\hat{v})}=\frac{\langle e({\bar{v}}^{E}+{\bar{v}}^{G}),{\bar{v}}^{G}\rangle }{\langle e({\hat{v}}^{E}+{\hat{v}}^{G}),{\hat{v}}^{G}\rangle }$$ (62)

where ${\bar{v}}^{E}$ and ${\bar{v}}^{G}$ represent the link flow patterns for EVs and GVs without tolls, ${\hat{v}}^{E}$ and ${\hat{v}}^{G}$ represent the link flow patterns for EVs and GVs with differentiable tolls.

**Proposition 5.** *Given a non-overlapping network*, *it holds that*:

$$POA_{E}\leq \frac{\eta_{\text{max}}}{\eta_{\text{min}}\left[1-{\left(\beta +1\right)}^{-1/\beta }-{\left(\beta +1\right)}^{-1}\right]}$$ (63)

**Prof.** See in Appendix 4.

The theoretical bound of *POA*_*E*_ is related to the parameter *β* in BPR function (9), *η*_*max*_ and *η*_*min*_ in Eq (11). It is proved that the upper bound of *POA*_*T*_ is independent on the urban network structure and percentage of electric vehicles.

We have algebraically derived out the general bound of *POA*_*T*_ and *POA*_*E*_, in order to validate the validity of Proposition 4 and Proposition 5, we will calculate the numerical bound of *POA*_*T*_ and *POA*_*E*_ by conducting case studies based on the actual network and travel demand of two cities in China: Shenzhen and Lasa.

### 5.2 Simulated bound of price of anarchy

#### 5.2.1 Input data

The travel demand data used in this paper are based on the resident trip survey (The latest 2018) conducted twice per decade during the past 20 years and a traffic big data platform integrating mobile phone, public transport, taxi, navigation system, GPS system, and traffic volume data provided by Shenzhen Urban Planning Traffic Center (SUPTC).

The survey data include both ‘roadside interviews’ and ‘home interviews, such as trip behavior, the nature and intensity of traffic, the freight structure, income, employment, etc. The surveys were well defined and divided into ‘zones’ so that the origins and destinations of trips could be geographically observed. The variables used [37] for the interviews are given in Table 3.

**Table 3:** Interview survey variables.

**Home-interview trip data**	Date of journey	origin address and time
	destination address and time	journey purpose
	mode of travel	parking cost
**Household data**	number of family members Gender; Age	family relationships Household income
	Economic activity status	occupations
	Vehicle ownership	Tenure and property type
**Roadside-interview**	date of journey, cordon	crossing point
	Vehicle type and occupancy	Origin and destination

Basic travel demand data were collected by conducting a one-year home-interview survey during the afternoon peak hour (18:00–19:00). In transportation planning, fixed-demand models are often used to predict traffic flows during morning and evening peak hours because travel demand during rush hours mainly consists of commuters and is thus regarded as fixed.

In this research, total travel demand in Shenzhen are divided according to different administrative districts to estimate travel demand and generate the OD matrix (Table 4). The original input data used for the OD matrix are shown in S2 Text. There are 491 OD pairs with fixed demand in Shenzhen (S2 File). The number of OD pairs in different districts is provided in Table 5.

**Table 4:** OD matrix for data obtained during the afternoon rush hour (18:00–19:00) in Shenzhen.

Origin	Destination									
	Luo hu	Fu tian	Nan shan	Yan tian	Bao an	Long gang	Guangming	Ping shan	Long hua	Da peng
**Luo hu**	20313	8240	1668	344	1630	5352	507	223	2284	420
**Fu tian**	8762	46127	11110	179	5835	4064	964	119	5465	356
**Nan shan**	861	4345	35740	66	10165	1202	789	71	2076	71
**Yan tian**	1986	720	449	2894	409	2181	175	167	912	1159
**Bao an**	895	3233	13014	78	137964	1571	8494	115	3910	76
**Long gang**	9263	5377	2861	1279	2385	102941	708	4012	14157	1384
**Guangming**	197	519	1553	31	6503	542	34838	29	2032	13
**Ping shan**	618	327	216	114	194	3064	17	22222	777	795
**Long hua**	1366	2541	1908	135	2345	4968	1273	145	44602	110
**Da peng**	316	259	124	309	88	683	12	314	179	4976

**Table 5:** The number of OD pairs in 10 districts.

	Luo hu	Fu tian	Nan shan	Yan tian	Bao an	Long gang	Guang ming	Ping shan	Long hua	Da peng
The number of OD pairs	36	50	59	18	89	106	29	23	58	23

**Fig 3:**
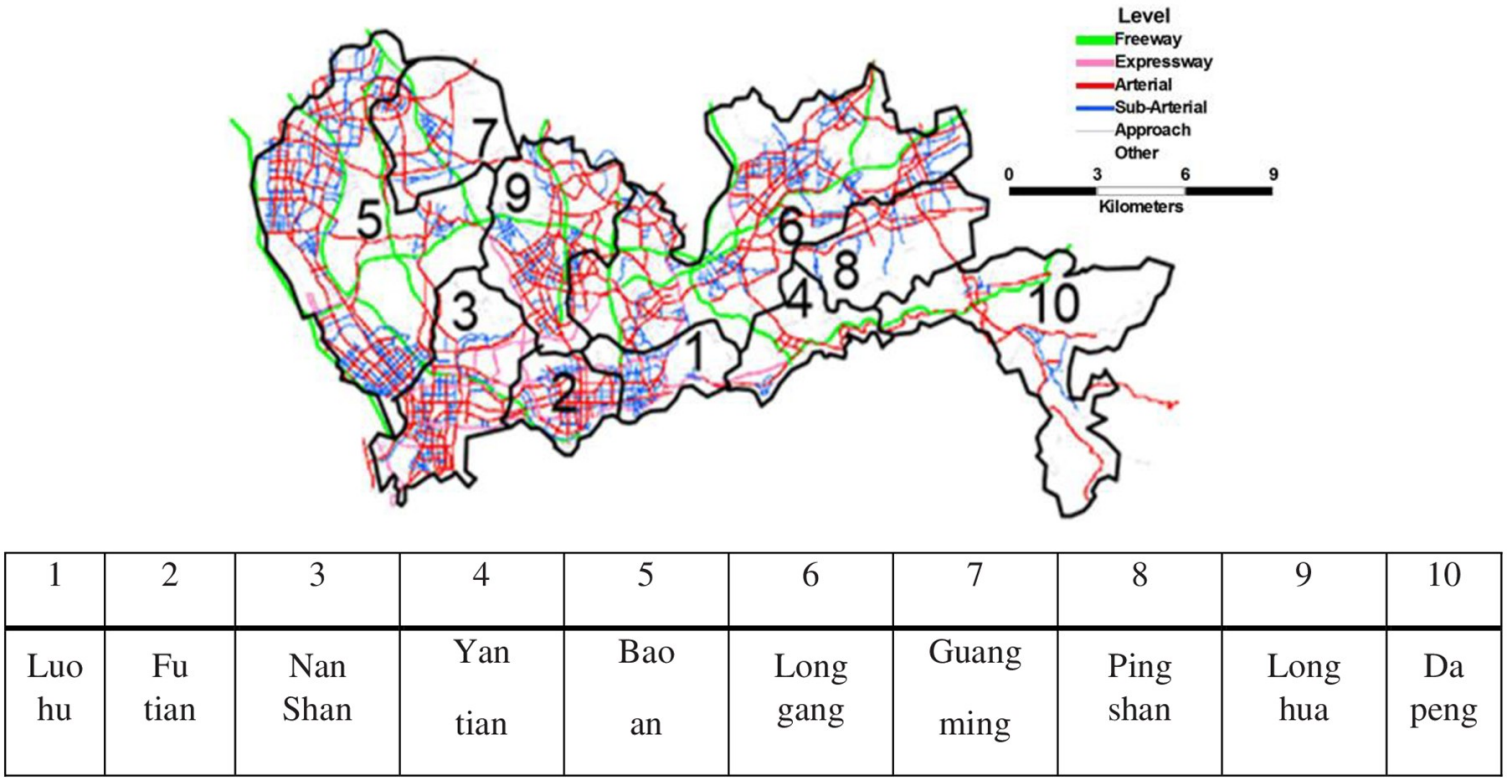
Reprinted from [Fig 3] under a CC BY license, with permission from [SUTC], original copyright [2018]. Network structure of Shenzhen considering different road types. Different colors represent different road types. The input data for Shenzhen are provided in S2 File.

The network topology and size are different in each district (Fig 3), and the number of links and nodes are summarized in Table 6.

**Table 6:** The network structures of the different districts.

	Luo hu	Fu tian	Nan Shan	Yan tian	Bao an	Long gang	Guangming	Ping shan	Long hua	Da peng
Link	7300	12742	14425	2386	33217	33217	7683	5054	13566	3045
Node	5171	8615	9835	1653	23263	21606	5460	3543	9613	2226

In general, there are 6 road types in Shenzhen, including expressways, arterial roads, sub arterial roads, and approach and side roads. These are represented by different colors in Fig 3, which displays the basic network structure and road types in Shenzhen.

The BPR link time function given in (9) is used in the case study, and the values of parameters **α**, *β* and $t_{a}^{0}$ of 6 different road types are shown in Table 7. The network structure in Shenzhen is shown in Fig 3.

**Table 7:** Parameters of the BPR link time functions for the different road types.

Road type	*α*	*β*	*C*	$t_{a}^{0}$
Freeway	1.5	2.1	2400	120
Expressway	1.4	2.2	2200	120
Arterial road	1.1	2.5	700	45
Subarterial road	1.2	2.6	500	35
Approach road	1.4	2.4	400	30
Side road	1.4	2.4	1000	45

The mileage of each road type in 10 districts of Shenzhen is given in Table 8:

**Table 8:** The number of different road types in 10 districts of Shenzhen.

Road type	Luo hu	Fu tian	Nan Shan	Yantian	Bao an	Long gang	Guangming	Ping shan	Long hua	Da peng
Freeway	8	18	32	31	154	152	58	21	61	41
Expressway	47	53	95	10	12	37	0	5	30	0
Arterial road	18	151	292	66	479	510	162	170	254	129
Subarterial road	96	104	138	9	422	296	84	86	139	33
Approach road	233	368	497	150	1216	1190	387	269	524	229
Side road	35	104	96	17	153	54	22	5	20	6

After preparing the data for conducting case study well, we will introduce the major procedure of the case study.

#### 5.2.2 Major procedure of case study

To calculate the numerical bounds of *POA*_*T*_ and *POA*_*E*_, this paper conducted a case study using the software TransCAD 6.0. The Geographic Information System Developer’s Kit (GISDK) [38] is a collection of software tools that come with TransCAD that makes it possible to feasibly revise the function in TransCAD software through integrating other programs. GISDK was used for the second development. The procedure includes the following steps:

Step 1: Given the road networks of Shenzhen, categorize all the links over the networks into 10 groups according to the districts;
Step 2: Use the OD table (Q) among 10 districts in Shenzhen to assign the total travel demand;
Step 3: Using the objective function file, where vs2010 compiling and customized DDL will be used, differentiable tolls are added to the link impedance function.

Then, we conduct the first case study based on 10 districts in Shenzhen and evaluate the efficiency gain of differentiable road pricing in managing congestion and vehicular emissions. The code of the following procedure is provided in S1 Text.

The major procedure used to simulate the Shenzhen networks

Step 1: Let *ϕ* = 0.25; multiply Q by *ϕ* to obtain the new ODtable *ϕ*Q

For *ϕ* = 0.25:2

  Step 2: Let *P* = 0; divide *Φ*Q into two groups: *ϕQ*_1_ (EV) and *ϕQ*_2_ (GV)

  For *P* = 0:1

  Step 3: Initialize the network

  Step 4: Create the net file for route selection and the net set

  Step 5: Traffic is assigned based on a certain objective function

  Step 6: Update the network file

  Step 7: Calculate the total system travel time and total system emissions across all links in each district. Save the output figures and the calculation results

  Step8: *P* = *P*+0.1

 End

Step9: Let *ϕ* = *ϕ* +0.25

End

7×9 simulations are conducted in each district, and 630 total simulations are conducted across the 10 networks of Shenzhen.

#### 5.2.3 Results analysis

*Results of* ***POA***_***T***_. Google Map is used to identify different road types in the road network of Shenzhen. The geographic information is input into a geographic information system (GIS), which is a software to compile the geographic information into a database and has the ability to illustrate the geographic information through graphs and charts. The input map data of Shenzhen are provided in S1 and S3 Files, S2 Text. Here, we choose the situation under original demand level (*ϕ = 1*) to display the distribution of total system travel time with and without road pricing. VOC represents the link travel cost varying from 0 (green) to 1.2 (red), which is represented by different colors. The number of link flows is represented by the line weight of the road link.

The results of case study show that both $T(\overset{-}{v})$ and $T(\hat{v})$ will not change with *P*, which is consistent with Lemma 1: by changing the percentage of EVs, *T*(**v**) remains constant.

**Fig 4:**
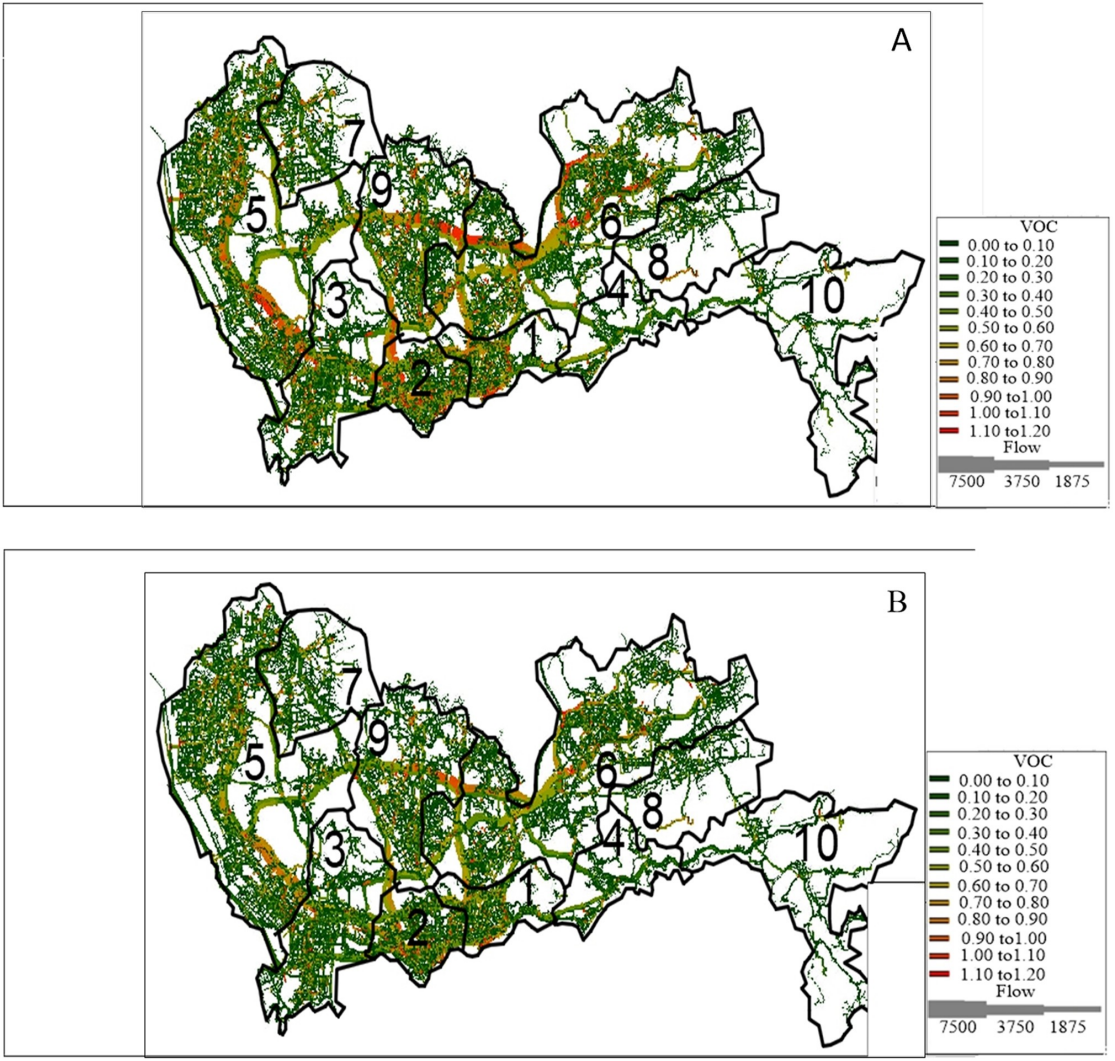
Reprinted from [Fig 4] under a CC BY license, with permission from [SUTC], original copyright [2018]. Distribution of total system travel time in Shenzhen. Fig 4 (A) displays $T(\overset{-}{v})$ without tolls (h), and Fig 4 (B) displays $T(\hat{v})$ with differentiable tolls (h).

After implementing differentiable tolls, the link-based trip distribution will change considerably, as shown in Fig 4. When *ϕ* = 1, the total travel time changes the least in Nanshan and the most in the Longhua district. The total travel time across 10 districts without tolls $T(\overset{-}{v})$ is 69083 h, and that with differentiable tolls $T(\hat{v})$ is 60767 h.

Since the travel demand data were collected by using a one-year home-interview survey during the afternoon peak hour, there are great differences in the congestion conditions during different time periods of the day. To better describe the variations in travel demand, the weighting factor of traffic demand level *ϕ* (multiplying the weighting factor by the original traffic demand) is introduced. Moreover, the percentage of EVs (*P*) among each OD pair with fixed demand is also introduced to simulate the mixed urban networks with both EVs and GVs.

In actual urban networks, the weighting factor of traffic demand level *ϕ* varies within a certain range. By changing *ϕ* within the range of 0.5~2, the results of link-based traffic flow solutions without and with differentiable tolls can be obtained (Table 9). It can be observed that both the total system travel time without tolls $T(\overset{-}{v})$ and the total system travel time with differentiable tolls $T(\hat{v})$ increase with the travel demand. It can be observed that when the demand level is 1, $T(\overset{-}{v})$ is the largest in Baoan district (15452 h), and $T(\hat{v})$ is the largest in Longgang district (13343 h); when the demand is doubled, both $T(\overset{-}{v})$ and $T(\hat{v})$ are the largest in Longgang district, at 42728 h and 34791 h, respectively. The results of case study (S1 Data) show that both $T(\overset{-}{v})$ and $T(\hat{v})$ do not change with the percentage of electric vehicles (EVs), which is consistent with Lemma 1. The results are shown in Table 9

**Table 9:** Total system travel time (h).

*ϕ*		0.5	0.75	1	1.25	1.5	1.75	2
Luohu	$T\left(\bar{v}\right)$	2232	3561	5080	6798	8813	11154	13842
	$T\left(\hat{v}\right)$	2039	3123	4309	5583	7341	9427	11725
Futian	$T\left(\bar{v}\right)$	3688	5960	8537	11555	15084	19099	23840
	$T\left(\hat{v}\right)$	3405	5256	7245	9429	11912	15383	19231
Nanshan	$T\left(\bar{v}\right)$	3461	5550	7877	11532	13328	16570	20193
	$T\left(\hat{v}\right)$	3443	5530	7868	10802	13213	16430	20025
Yantian	$T\left(\bar{v}\right)$	3461	1070	1549	2078	2689	3375	4165
	$T\left(\hat{v}\right)$	3298	1057	1372	1552	2599	3161	3842
Baoan	$T\left(\bar{v}\right)$	7134	11089	15452	20289	25697	31709	38381
	$T\left(\hat{v}\right)$	6556	9724	13167	16598	20465	25495	30715
Longgang	$T\left(\bar{v}\right)$	6677	10676	15311	20783	27169	34407	42728
	$T\left(\hat{v}\right)$	6189	9547	13343	17571	21800	28645	34791
Guangming	$T\left(\bar{v}\right)$	1540	2436	3434	4502	4502	7881	9627
	$T\left(\hat{v}\right)$	1513	2355	3423	5236	4065	7025	8495
Pingshan	$T\left(\bar{v}\right)$	704	1145	1659	2940	2940	3699	4540
	$T\left(\hat{v}\right)$	689	1099	1548	2397	2619	3224	3904
Longhua	$T\left(\bar{v}\right)$	3760	6274	9219	16551	16551	20848	25932
	$T\left(\hat{v}\right)$	3345	5320	7561	13158	13000	17252	21565
Dapeng	$T\left(\bar{v}\right)$	443	443	965	1271	1597	1967	2378
	$T\left(\hat{v}\right)$	429	434	931	974	1504	1837	2190

After obtaining the calculation results of $T\left(\bar{v}\right)$ and $T\left(\hat{v}\right)$, the results of *POA*_*T*_ can be calculated according to Eq (60). By changing the weighting factor of demand level *ϕ* from 0.5 to 2, the results of *POA*_*T*_ under different *ϕ* can be obtained. The results are summarized in Table 10.

**Table 10:** The calculation results of *POA*_*T*_ in 10 districts.

*∅*	Luo Hu	Fu tian	Nan Shan	Yan tian	Bao an	Long gang	Guangming	Ping shan	Long hua	Da peng
0.5	1.09453	1.08297	1.00535	1.04941	1.08816	1.07877	1.01764	1.0218	1.12397	1.03182
0.75	1.14041	1.13404	1.00361	1.01215	1.14042	1.11829	1.03421	1.04189	1.1794	1.02041
1	1.17894	1.1784	1.00115	1.12906	1.17357	1.14748	1.05142	1.07175	1.21936	1.03646
1.25	1.21768	1.22545	1.06754	1.33927	1.22241	1.18278	1.2012	1.22649	1.25786	1.30439
1.5	1.20054	1.26626	1.00871	1.03459	1.25566	1.24626	1.10757	1.12252	1.27314	1.0616
1.75	1.18324	1.24153	1.00854	1.06756	1.24372	1.20115	1.12187	1.14724	1.20844	1.07085
2	1.18052	1.23969	1.00841	1.08401	1.2496	1.22814	1.13325	1.16295	1.20248	1.08562

As shown in Table 10, the upper numerical bound of *POA*_*T*_ is 1.3393. When *Φ* is 0.5, 0.75, 1 and 1.5, the upper bound of *POA*_*T*_ occurs in the Longhua district; when *Φ* is 1.25, the upper bound of *POA*_*T*_ occurs in Yantian. When *Φ* is 1.75 and 2, the upper bound of *POA*_*T*_ occurs in Baoan. The upper bounds of *POA*_*T*_ under different *Φ* in each district is shown in Table 11.

**Table 11:** Upper bound of *POA*_*T*_ in 10 districts.

District	Luo hu	Fu tian	Nan Shan	Yan tian	Bao an	Long gang	Guang ming	Ping shan	Long hua	Da peng
$POA_{T}{}_{{}_{max}}$	1.218	1.266	1.068	1.339	1.256	1.246	1.201	1.227	1.273	1.304

The variation regulation of *POA*_*T*_ in accordance with *Φ* is similar across 10 districts and first increases and then decreases, reaching the maximum value when the value of *Φ* is between 1.2 and 1.5. All 10 districts show a similar trend, which implies that there must be a mechanism that drives this regulation. It can also be concluded that the variation in *POA*_*T*_ in terms of *Φ* is independent on the network structure. The results of case study show that the upper bound of *POA*_*T*_ across 10 districts is **1.3393 (**Table 11**)**.

**Fig 5:**
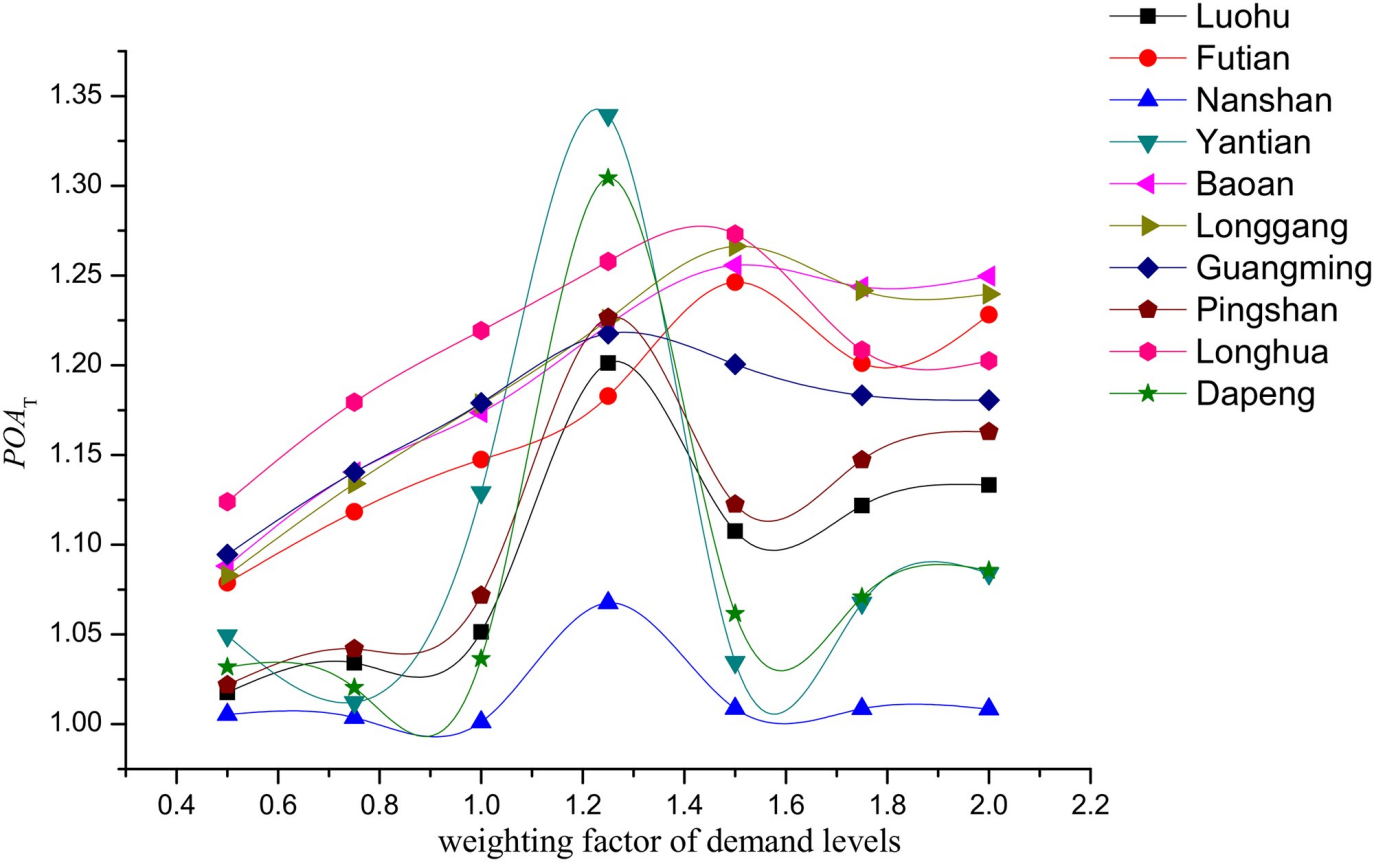
Relationship between *POA*_*T*_ and demand level *Φ*. Each curve represents the variation of *POA*_*T*_ in terms of *Φ* in the 10 districts of Shenzhen.

The value of *POA*_*T*_ in Nanshan is always the smallest among the 10 districts, implying that the travelers in this district are the least sensitive to the implementation of the differentiable road pricing scheme (Fig 5). Since Nanshan is not only the most economically powerful district in Shenzhen but also the center of scientific research, business, enterprise, and education, travelers commuting from Nanshan are less sensitive to the charging road pricing.

If we focus on the maximum value of each curve, the maximum *POA*_*T*_ is in Luo Hu, while Futian and Nanshan have the lowest values among 10 districts. These three districts are within the boundaries of the Shenzhen Special Economic Zone (SEZ), in which both the GDP and population rank among the top 3 in Shenzhen. It is showed that the efficiency gain of differentiable road pricing in the districts within the SEZ is less than that outside of the SEZ.

#### Results of POA_E_

**Fig 6:**
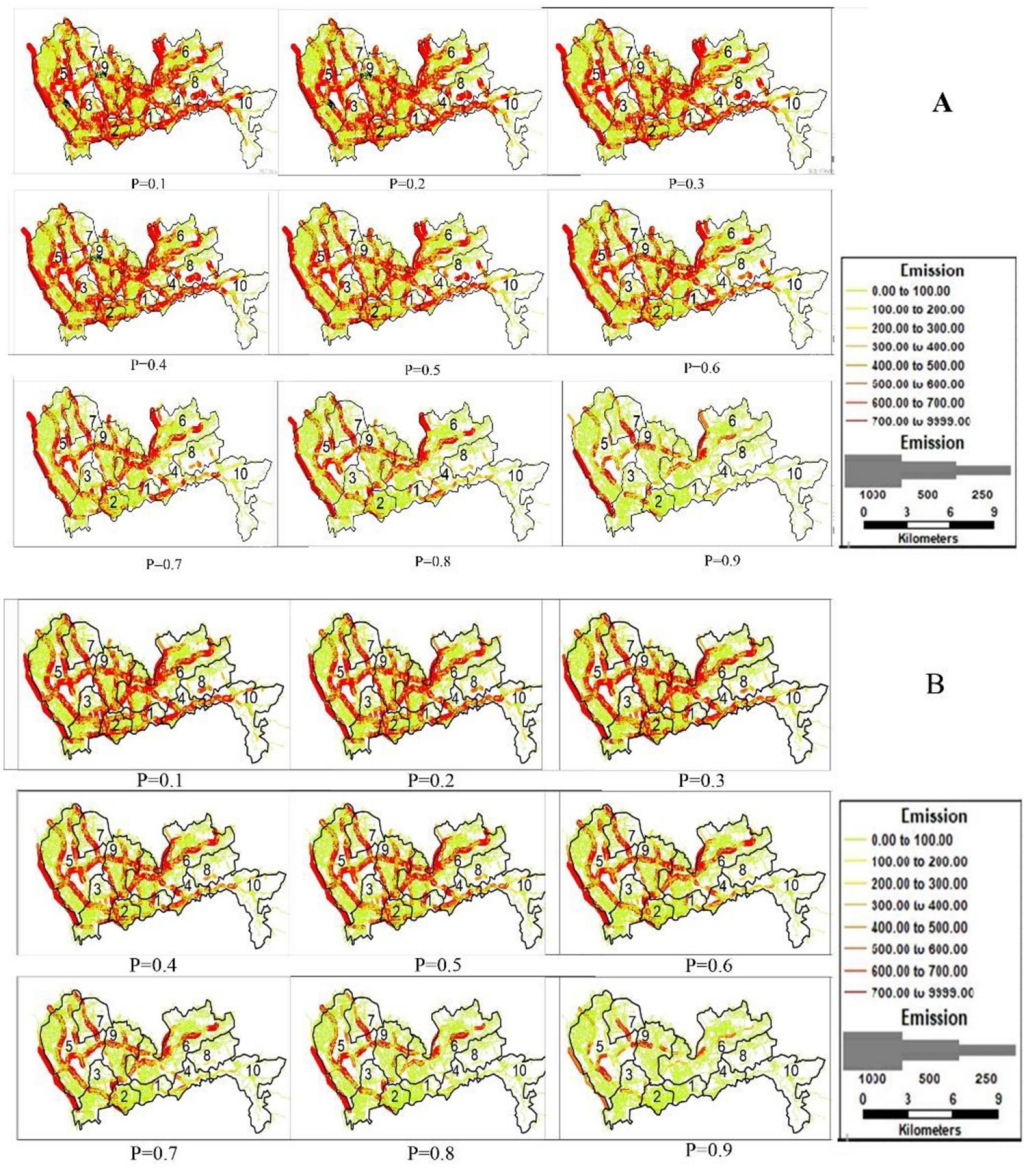
Reprinted from [Fig 6] under a CC BY license, with permission from [SUTC], original copyright [2018] total system emissions. Distribution of emissions in Shenzhen. Fig 6 (A) displays $E(\bar{v})$ without tolls (g/h), and Fig 6 (B) displays $E(\hat{v})$ with differentiable tolls (g/h).

When the percentage of EVs (*P*) continuously changes from 0.1 to 0.9, the distribution of vehicular emissions under scenarios without and with differentiable tolls are shown in Fig 6.

Here, we display the results of $E(\bar{v})$ and $E(\hat{v})$ under original demand level (***Φ* = 1**). It is shown that the differentiable road pricing scheme could efficiently manage vehicular emissions.

**Fig 7:**
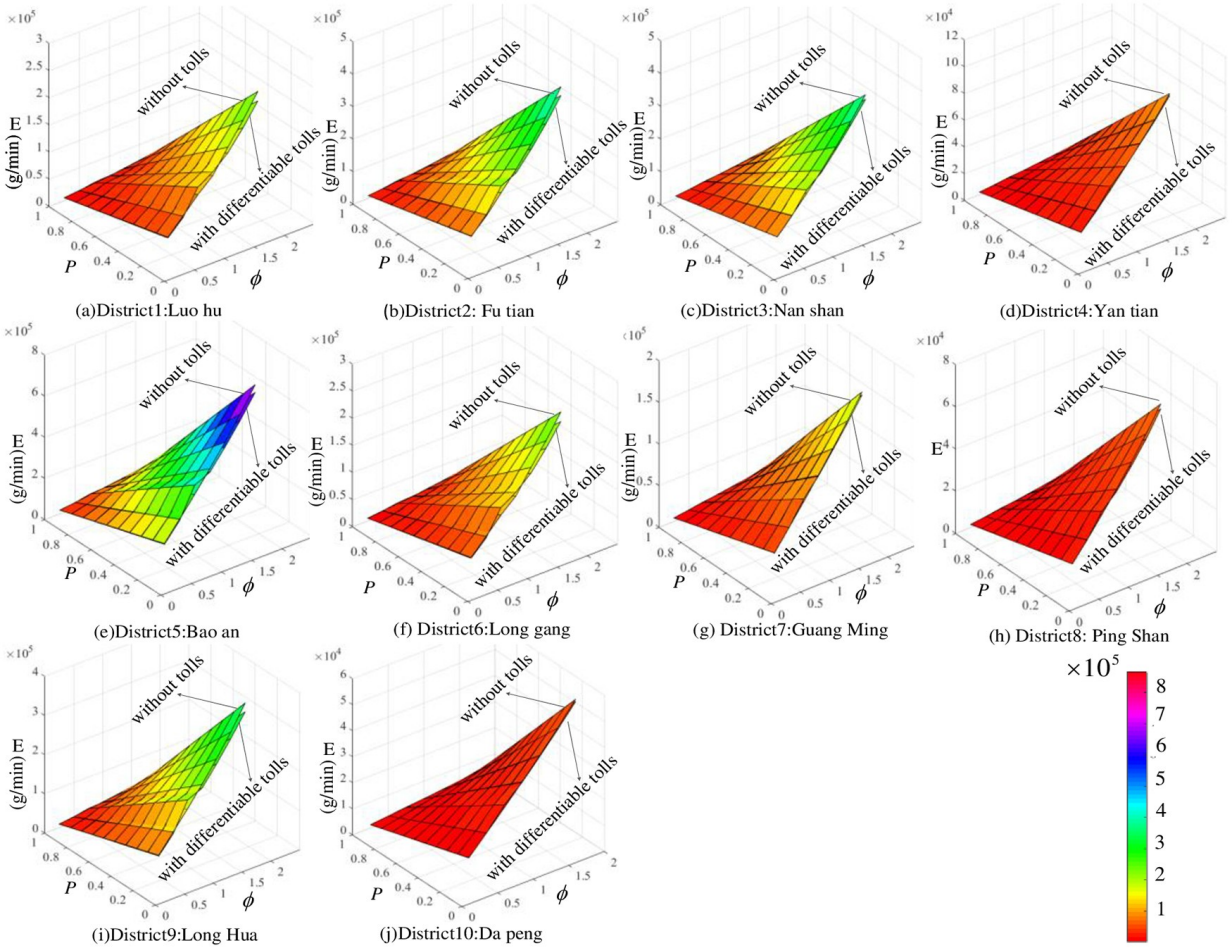
*E*-*P*-*ϕ* surf graph. Fig 7 (*a*)-(*j*) illustrates the relationship among *E*-*P*-***ϕ*** in different districts; the upper layer represents $E(\bar{v})$ without tolls (g/h), and the lower layer represents $E(\hat{v})$ with differentiable tolls (g/h).

Unlike the calculation results of the total system travel time, where both $T(\overset{-}{v})$ and $T(\hat{v})$ do not change with *P* (Fig 4), the results in Fig 6 show that both $E(\bar{v})$ and $E(\hat{v})$ decrease with *P*. It is shown in Fig 6 (A) and S1 Data that $E(\bar{v})$ will decrease from 1778098 g/h to 175342 g/h when *P* increases from 0 to 0.9. After implementing the differentiable tolls, $E(\hat{v})$ will decrease from 1647737 g/h to 162181 g/h with an increase in *P* (Fig 6 (A)). If we fix *P*, the surface of $E(\bar{v})$ is always above that of $E(\hat{v})$. Take a look at the case when *P* is 0.5, the result of $E(\bar{v})$ and $E(\hat{v})$ is 887407 g/h and 822227 g/h, respectively. When ***Φ* = 1**, both $E(\bar{v})$ and $E(\hat{v})$ change with *P* (Table 12). The other cases are shown in Fig 7 and S1 Data.

**Table 12:** $E(\bar{\text{v}})$ and $E(\hat{\text{v}})$ under *Φ* = 1 (g/h).

*P*	0	0.1	0.2	0.3	0.4	0.5	0.6	0.7	0.8	0.9
$E(\bar{v})$	1778098	1599490	1421450	1243435	1065409	887407	709373	531337	353344	175342
$E(\hat{v})$	1647737	1482343	1317314	1152273	987244	822227	657199	492172	327161	162181

According to the calculation results displayed in Fig 7, there are more vehicular emissions in Luohu, Futian, Nanshan, Baoan and Longhua than in the other 5 districts. After implementing the differentiable road pricing scheme, total system emissions are reduced in each district to an extent. The upper surface shown in Fig 7 represents total emissions without tolls, and the lower curve surface represents total system emissions with differentiable tolls. It is shown that differentiable tolls can efficiently decrease total system emissions. There are more vehicular emissions in Luohu, Futian, Nanshan, Baoan and Longhua than in the other 5 districts. All 10 networks in Shenzhen display a similar trend: If we fix *P*, *E* will increase with *ϕ*; if we fix *ϕ*, *E* will decrease with *P*.

After obtaining the calculation results of $E(\bar{v})$ and $E(\hat{v})$, the numerical results of *POA*_*E*_ can be obtained according to formula (62), which is provided in S3 Data. The upper bound of *POA*_*E*_ across the 10 districts in Shenzhen is **1.4414**. The values of *POA*_*E*_ in different districts always reach a maximum when *Φ* is 1.25 and *P* is 0.9. The numerical upper bound of *POA*_*E*_ in each district of Shenzhen is shown in Table 13. The calculation results of *POA*_*E*_ can be obtained by multiplying the original matrices by different *ϕ*. Here, *ϕ* changes from 0.5 to 2 and *P* changes from 0.1 to 0.9; 7×9 simulations are carried out in each district, and 630 simulations are carried out across the 10 networks in Shenzhen.

**Table 13:** Numerical upper bound of *POA*_*E*_ in the 10 districts.

District *POA*_*E*_	Luo hu	Fu tian	Nan shan	Yan tian	Bao an	Long gang	Guang ming	Ping shan	Long hua	Da peng
Max	1.064	1.037	1.009	1.054	1.042	1.003	1.003	1.009	1.020	1.0077

**Fig 8:**
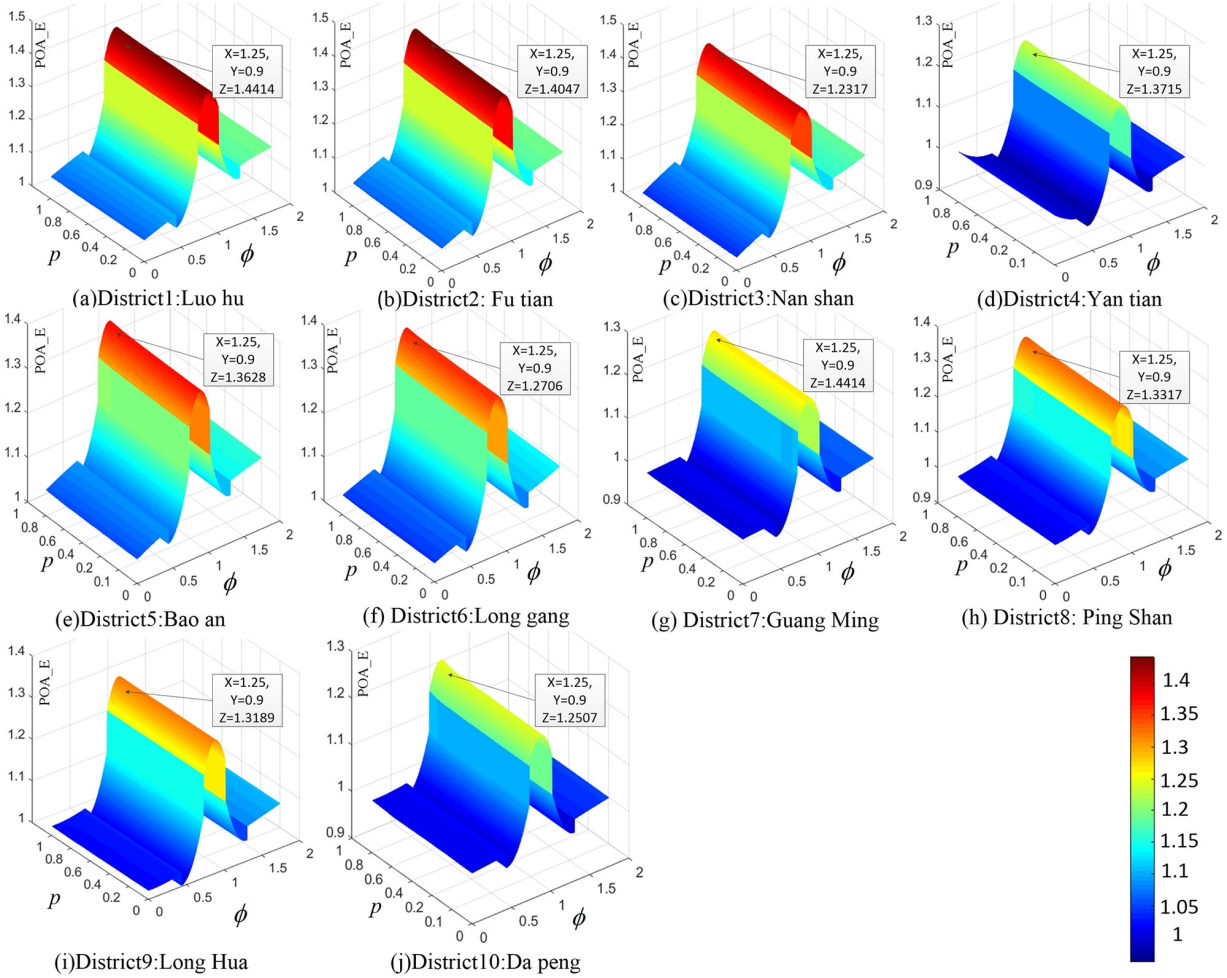
*P*-*Φ*-*POA*_*E*_ surface graph. Fig 8 (*a*)- Fig 6 (*j*) illustrate the relationships among *P*, *Φ* and *POA*_*E*_ in the different districts of Shenzhen.

As is shown in S3 Data, the percentage of electric vehicles (*P*) has direct impacts on total system emissions (*E*) but has little impact on the value of *POA*_*E*_. To draw out the smooth surface graph shown in Fig 8, each matrix with 7×9 dimensions can be expanded into 350×450 dimensions through the cubic spline interpolation method. The *P*-*Φ*-*POA*_*E*_ surface graph (Fig 8) is plotted to show the relationship among *P*, *Φ* and *POA*_*E*_.

The similarities across the 10 networks in Shenzhen imply that there must be general mechanisms to explain the variations. However, the individual data of each district show that the values of *POA*_*E*_ are very different in each district, and they are too varied to be statistically significant. For example, Fig 8(*d*) has only 2 peaks, while the figures for other districts have 3 peaks considering the relationship between *POA*_*E*_ and *Φ*. Above all, the variation trends of *POA*_*E*_ regarding *Φ* and *P* tend to be similar across 10 networks in Shenzhen.

We also have some interesting findings from the simulation results: the variation trends of *POA*_*T*_ and *POA*_*E*_ in terms of *ϕ* are similar across the different networks, implying that these values are independent of the network topology. The efficiency gain of differentiable road pricing in managing system total travel time is less in the districts within the Shenzhen Economic Special Zone (SEZ) than in the other districts, and the efficiency gain of differentiable road pricing in managing system total emissions is larger in the districts within the SEZ than in the other districts, which implies that differentiable road pricing is more effective for managing congestion outside the SEZ and managing emissions within the SEZ.

#### 5.2.5 Feasibility validation

*1) Confirmation experiment*. Proposition 4 and Proposition 5 indicate that the bound of price of anarchy for total system travel time (*POA*_*T*_) and (*POA*_*E*_) are independent on the network structure, and these regulations observed on the 10 networks of Shenzhen also exist on networks of other cities. In order to validate the theoretical bound and generality of these insights and findings, we conducted another case study based on a city in China, Lasa. This case study is conducted based on the same procedure introduced before.

**Fig 9:**
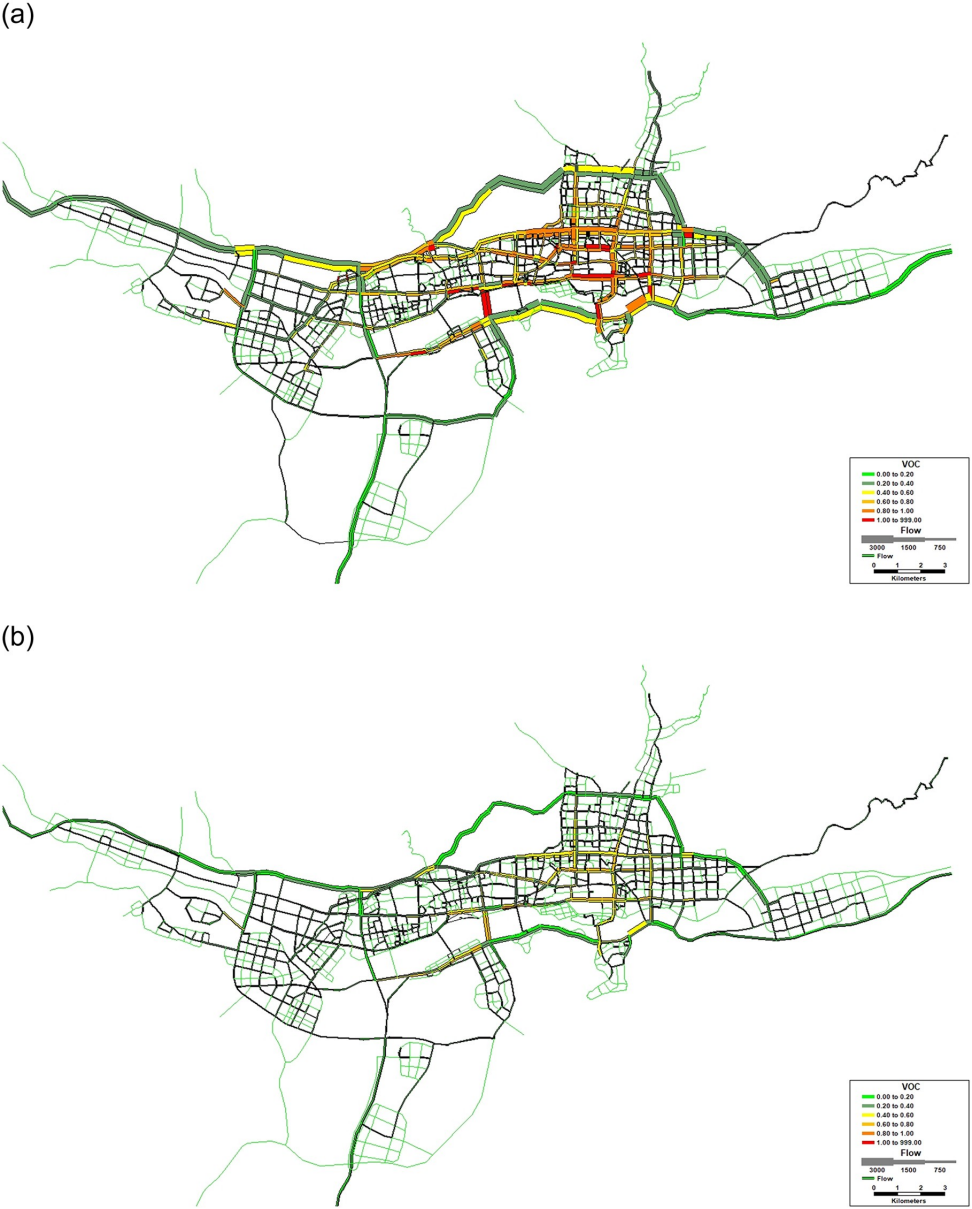
Reprinted from [Fig 9] under a CC BY license, with permission from [SUTC], original copyright [2018]. Distribution of total system travel time in Lasa. Fig 9 (A) displays $T(\bar{v})$ without tolls (h), and Fig 9 (B) displays $T(\hat{v})$ with differentiable tolls (h).

- Results of Total system travel time Fig 9

Likewise, we only choose the scenario with original demand level (*ϕ* = 1) to display the link-based traffic flow pattern without differentiable tolls $\overset{-}{v}$, and link-based traffic flow pattern with differentiable tolls $\hat{v}$, respectively, and other results can be found in S2 Data.

Similarly, the total system travel time decreases a lot after implementing the differentiable road pricing scheme. Then we display the results of only $E(\overset{-}{v})$ and $E(\hat{v})$ for the original demand level (*ϕ* = 1). After obtaining the calculation results of $T(\overset{-}{v})$ and $T(\bar{v})$, the results of *POA*_*T*_ can be calculated according to Eq (60). By changing the weighting factor of demand level *Φ* from 0.5 to 2, the calculation results of *POA*_*T*_ under different *Φ* can be obtained. The results are summarized in Table 14.

**Table 14:** The calculation results of *POA*_*T*_ in Lasa.

*Φ*	0.5	0.75	1	1.25	1.5	1.75	2
*POA*_*T*_	1.0801	1.1304	1.1679	1.2287	1.2653	1.2134	1.2161

**Fig 10:**
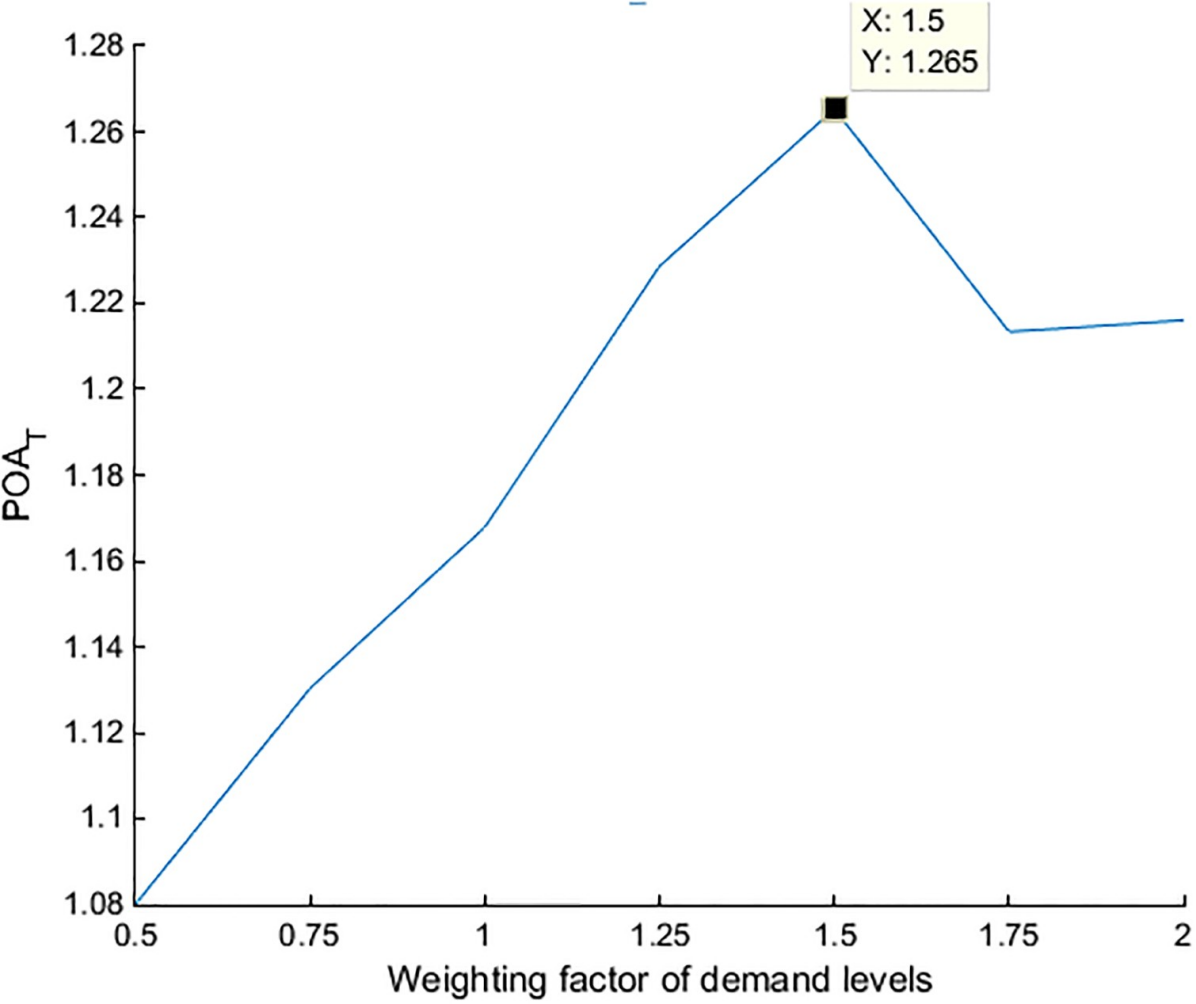
Relationship between *POA*_*T*_ and demand level *ϕ*.

As is shown in Fig 10 and Table 14, the upper numerical bound of *POA*_*T*_ in Lasa is **1.2653** when the value of *Φ* is 1.5. Similar to the calculation results of Shenzhen (Table 10), the numerical bound of *POA*_*T*_ in Lasa is bounded by the theoretical bound in Proposition 4. The variation regulation of *POA*_*T*_ in Lasa is similar to that in Shenzhen, which validates that the bound of *POA*_*T*_ is independent on the urban traffic network structure. and other results can be found in S2 Data.

- Results of total system emissions

**Fig 11:**
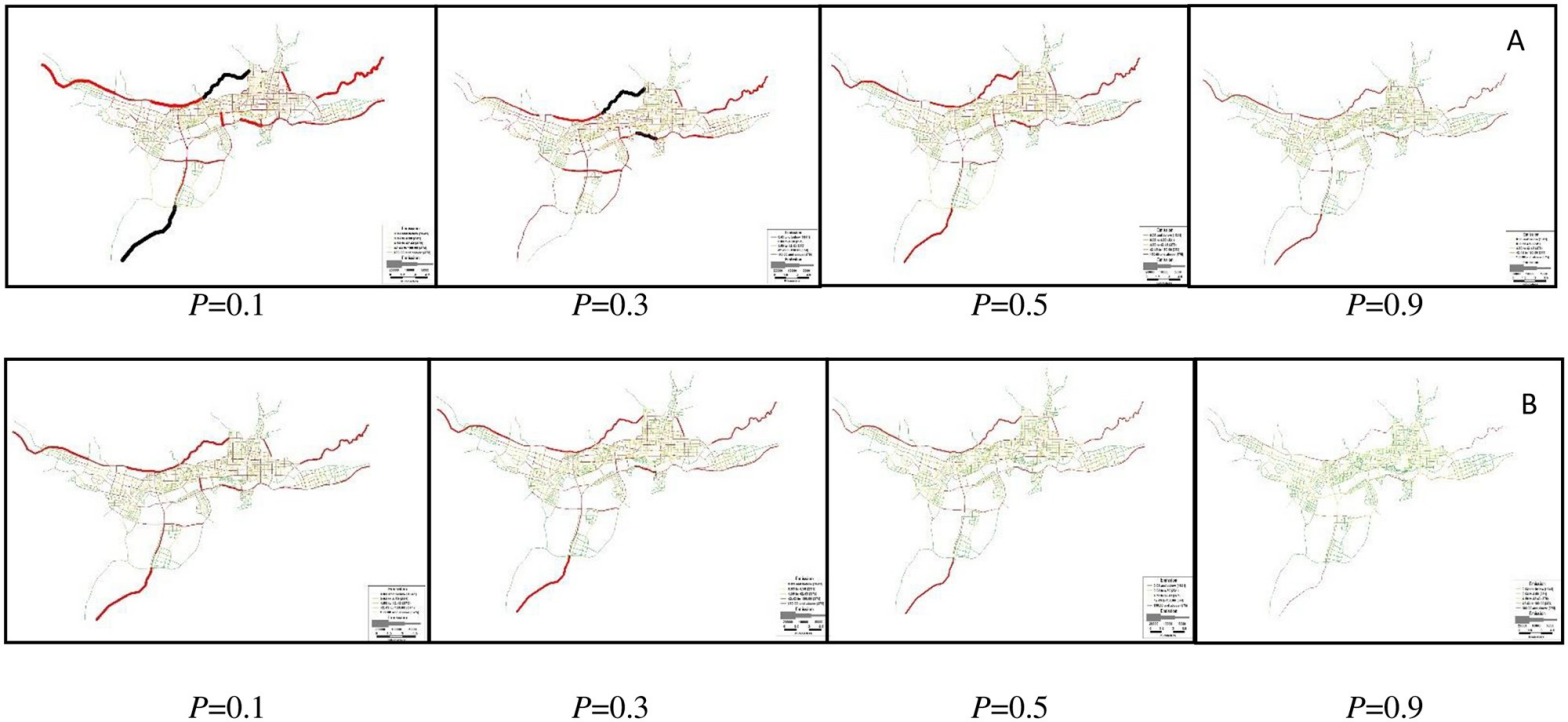
Reprinted from [Fig 11] under a CC BY license, with permission from [SUTC], original copyright [2018]. Distribution of total system emissions in Lasa. Fig 11 (A) displays $E(\bar{v})$ without tolls (g/h), and Fig 11 (B) displays $E(\hat{v})$ with differentiable tolls (g/h).

In Fig 11, if we fix the percentage of EVs (*P*) and compare Fig 11(A) with Fig 11(B) respectively, it is showed that the total system emissions in B are always smaller than that in A, indicating that the differentiable road pricing scheme could efficiently manage vehicular emissions. If we observe Fig 11(A) and 11(B), total system emissions decrease with *P* in both cases.

**Fig 12:**
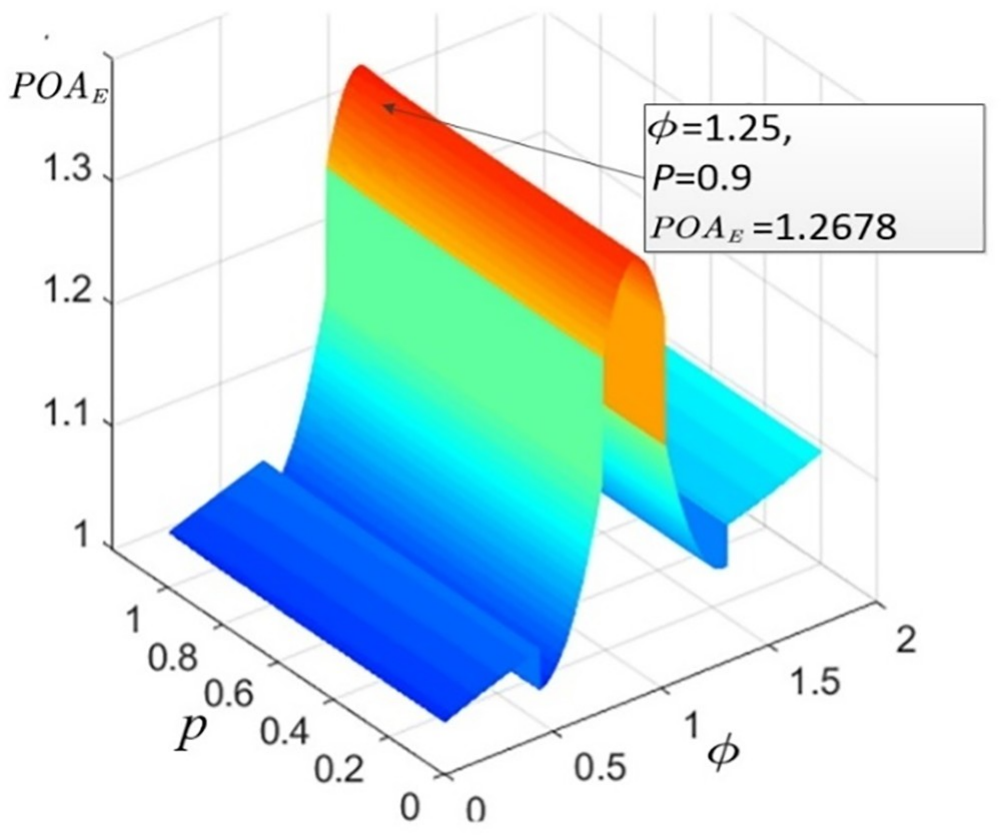
*P*-*Φ*-*POA*_*E*_ surface graph.

After obtaining the calculation results of $E(\bar{v})$ and $E(\hat{v})$, the numerical results of *POA*_*E*_ can be obtained according to formula (62), which is provided in S4 Data. The upper numerical bound of *POA*_*E*_ in Lasa is **1.2678** when *Φ* is 1.25 and *P* is 0.9. the variation trends of *POA*_*E*_ regarding *Φ* and *P* tend to be similar with Shenzhen (Fig 12).

*2) Sensitivity analysis on theoretical bound of POA*_*T*_. As is shown in Proposition 4, theoretical bound of *POA*_*T*_ is only related to the parameter *β* in BPR function, the value of parameter *β* is determined by the road type (Table 7). The calculation results show that the numerical upper bound of *POA*_*T*_ in Shenzhen is **1.3393**, and the numerical upper bound of *POA*_*E*_ in Lasa is **1.2653**. According to Table 7, the value of *β* is decided by the road type and can be set within the range of 2.1–2.6. Then we substitute the value of *β* into Proposition 4 and conduct parameter analysis on the values of ${POA}_{T}^{*}$ (Table 15).

**Table 15:** Theoretical bound of ${POA}_{T}^{*}$.

*β*	2.1	2.2	2.5	2.6	2.4	2.4
$POA_{T}^{*}$	1.654	1.681	1.763	1.790	1.736	1.736

Sensitivity analysis on parameter *β* (Table 15) show that the numerical values of *POA*_*T*_ can always be bounded by ${POA}_{T}^{*}$, this analysis validate the effectiveness of Proposition 4.

*3) Sensitivity analysis on theoretical bound of POA*_*E*_. As is shown in Proposition 5, theoretical bound of *POA*_*E*_ is related to the parameter *β*, the percentage of EVs *P*, the upper and lower bound of emission factor, *η*_*max*_ and *η*_*min*_. Then we substitute all of values of parameters into Proposition 4 and conduct parameter analysis on the values of ${POA}_{E}^{*}$ (Table 16). The calculation results show that the numerical upper bound of *POA*_*E*_ in Shenzhen is **1.3393**, and the numerical upper bound of *POA*_*E*_ in Lasa is **1.2678**. According to Table 7, the value of *β* is decided by the road type and can be set within the range of 2.1–2.6.

**Table 16:** Theoretical bound of ${POA}_{E}^{*}$.

*β*	2.2	2.3	2.4	2.5	2.6
$POA_{E}^{*}$	1.4705	1.5510	1.6275	1.7004	1.7698

Sensitivity analysis on parameter *β* (Table 16) show that the numerical values of *POA*_*T*_ can always be bounded by ${POA}_{E}^{*}$, this analysis validate the effectiveness of Proposition 5.

From the experimental results of both cities, the variation trends of *POA*_*T*_ and *POA*_*E*_ are similar across the different networks, implying that the bound of price of anarchy is independent on the network structure and percentage of electric vehicles.

## 6 Conclusions and future study

In this paper, we theoretically and empirically bound the efficiency of differentiable road pricing for EVs and GVs. Considering the asymmetric link flow interactions between EVs and GVs, Proposition 1 and Proposition 2 prove that the differentiable road pricing can decentralize a Pareto-efficient flow as the unique user equilibrium. The deviation gap between the single-criterion-based system optimal on Pareto frontier is bounded by two deviation factors and given in Lemma 1 and Proposition 3. Finally, the theoretical bound of *POA*_*T*_ and *POA*_*E*_ is given in Proposition 4 and Proposition 5. In order to validate the effectiveness of theoretical bound, we conduct case studies based on two cities of China: Shenzhen and Lasa. Empirical results show that the numerical bound of *POA*_*T*_ is **1.3393** in Shenzhen and **1.2653** Lasa, respectively. The numerical bound of *POA*_*E*_ is **1.4414** in Shenzhen and **1.2678** in Lasa, respectively. After sensitivity analysis on different parameters related to the bound of POA, we validate the effectiveness of Proposition 4 and Proposition 5. Moreover, the variation regulation of POA experiences a similar trend in different networks, which implies that there must be a mechanism driving this regulation. From both theoretical derivation and empirical analysis, it can be concluded that the bound *POA*_*T*_ in is independent on the network structure and percentage of electric vehicles. To develop this research further, the model will be extended to the multi-class model with elastic demand, where users’ various value of time and travel demand will be considered. This model can also be extended to include other air pollutants except for carbon monoxide (CO).

## Appendix

### Appendix 1. Jacobian matrix of ∇_v_*t*(v) is a ∇_v_*e*(v)

$$\nabla_{v}t\left(v\right)=\left[\begin{array}{ccccccc} \frac{\partial t_{1}^{E}\left(v\right)}{v_{1}^{E}} & \frac{\partial t_{1}^{E}\left(v\right)}{v_{1}^{G}} & \frac{\partial t_{1}^{E}\left(v\right)}{v_{2}^{E}} & \frac{\partial t_{1}^{E}\left(v\right)}{v_{2}^{G}} & \cdots & \frac{\partial t_{1}^{E}\left(v\right)}{v_{n}^{E}} & \frac{\partial t_{1}^{E}\left(v\right)}{v_{n}^{G}}\\ \frac{\partial t_{1}^{G}\left(v\right)}{v_{1}^{E}} & \frac{\partial t_{1}^{G}\left(v\right)}{v_{1}^{G}} & \frac{\partial t_{1}^{G}\left(v\right)}{v_{2}^{E}} & \frac{\partial t_{1}^{G}\left(v\right)}{v_{2}^{G}} & \cdots & \frac{\partial t_{1}^{G}\left(v\right)}{v_{n}^{E}} & \frac{\partial t_{1}^{G}\left(v\right)}{v_{n}^{G}}\\ \frac{\partial t_{2}^{E}\left(v\right)}{v_{1}^{E}} & \frac{\partial t_{2}^{E}\left(v\right)}{v_{1}^{G}} & \frac{\partial t_{2}^{E}\left(v\right)}{v_{2}^{E}} & \frac{\partial t_{2}^{E}\left(v\right)}{v_{2}^{G}} & \cdots & \frac{\partial t_{2}^{E}\left(v\right)}{v_{n}^{E}} & \frac{\partial t_{2}^{E}\left(v\right)}{v_{n}^{G}}\\ \frac{\partial t_{2}^{G}\left(v\right)}{v_{1}^{E}} & \frac{\partial t_{2}^{G}\left(v\right)}{v_{1}^{G}} & \frac{\partial t_{2}^{G}\left(v\right)}{v_{2}^{E}} & \frac{\partial t_{2}^{G}\left(v\right)}{v_{2}^{G}} & \cdots & \frac{\partial t_{2}^{G}\left(v\right)}{v_{n}^{E}} & \frac{\partial t_{2}^{G}\left(v\right)}{v_{n}^{G}}\\ \vdots & \vdots & \vdots & \vdots & \cdots & \vdots & \vdots \\ \frac{\partial t_{n}^{E}\left(v\right)}{v_{1}^{E}} & \frac{\partial t_{n}^{E}\left(v\right)}{v_{1}^{G}} & \frac{\partial t_{n}^{E}\left(v\right)}{v_{2}^{E}} & \frac{\partial t_{n}^{E}\left(v\right)}{v_{2}^{G}} & \cdots & \frac{\partial t_{n}^{E}\left(v\right)}{v_{n}^{E}} & \frac{\partial t_{n}^{E}\left(v\right)}{v_{n}^{G}}\\ \frac{\partial t_{n}^{G}\left(v\right)}{v_{1}^{E}} & \frac{\partial t_{n}^{G}\left(v\right)}{v_{1}^{G}} & \frac{\partial t_{n}^{G}\left(v\right)}{v_{2}^{E}} & \frac{\partial t_{n}^{G}\left(v\right)}{v_{2}^{G}} & \cdots & \frac{\partial t_{n}^{G}\left(v\right)}{v_{n}^{E}} & \frac{\partial t_{n}^{G}\left(v\right)}{v_{n}^{G}} \end{array}\right]$$

$$\nabla_{v}e\left(v\right)=\left[\begin{array}{ccccccc} 0 & \frac{\partial e_{1}^{G}\left(v\right)}{v_{1}^{G}} & 0 & \frac{\partial e_{1}^{G}\left(v\right)}{v_{2}^{G}} & \cdots & 0 & \frac{\partial e_{1}^{G}\left(v\right)}{v_{n}^{G}}\\ 0 & \frac{\partial e_{1}^{G}\left(v\right)}{v_{1}^{G}} & 0 & \frac{\partial e_{1}^{G}\left(v\right)}{v_{2}^{G}} & \cdots & 0 & \frac{\partial e_{1}^{G}\left(v\right)}{v_{n}^{G}}\\ \vdots & \vdots & \vdots & \vdots & \vdots & \ddots & \frac{\partial e_{1}^{G}\left(v\right)}{v_{n}^{G}}\\ 0 & \frac{\partial e_{1}^{G}\left(v\right)}{v_{1}^{G}} & 0 & \frac{\partial e_{1}^{G}\left(v\right)}{v_{1}^{G}} & \cdots & 0 & \frac{\partial e_{1}^{G}\left(v\right)}{v_{n}^{G}} \end{array}\right]$$

### Appendix 2. Proof of Lemma 1

**Proof.** If *P* = 0, the upper bound of total system emissions *E*(v) is *η*_*max*_*t*_*a*_(*v*_*a*_) · *v*_*a*_. If *P* = 1, there are no emissions. If 0<*P*<1, the lower bound of total system emissions is (1 − *P*)*η*_*max*_*t*_*a*_(*v*_*a*_)*v*_*a*_, and thus, we obtain inequality (64):

$$\frac{T\left(v\right)}{E\left(v\right)}\leq \frac{1}{\left(1-P\right)\eta_{\text{min}}}$$ (64)

Next, we derive the upper bound of total system emissions under differentiable tolls. Consider the following minimization program:

$$\underset{f}{\text{min}}\sum_{a\in A}e_{a}\left(v_{a}\right)\cdot v_{a}^{G}$$ (65)

which is subject to constraints (21)-(25).

According to dual theory, at the optimal point, we have:

$$\sum_{a\in A}v_{a}^{G}\lambda_{a}^{G}=\sum_{a\in A}e_{a}\left(v_{a}\right)\cdot v_{a}^{G}-\sum_{w\in W}d_{w}^{G}\mu_{w}^{G}$$ (66)

According to Eq (32), we have:

$$\sum_{a\in A}\left[t_{a}\left(v_{a}\right)-\lambda_{a}^{E}\right]\delta_{ar}^{w,E}=\mu_{w}^{E},w\in W,r\in R_{w}$$ (67)

By multiplying both sides of Eq (67) by $\sum_{r\in R_{w}}f_{rw}=d_{w}$ and summarizing all OD pairs *w* ∈ *W*, we obtain:

$$\sum_{a\in A}t_{a}\left(v_{a}\right)\cdot v_{a}=\sum_{a\in A}\lambda_{a}^{E}v_{a}+\sum_{w\in W}\mu_{w}^{E}d_{w}$$ (68)

By multiplying both sides of (68) with *P*, we obtain:

$$P\sum_{a\in A}t_{a}\left(v_{a}\right)\cdot v_{a}\leq \sum_{w\in W}\mu_{w}^{E}d_{w}^{E}$$ (69)

First, we multiply both sides of inequality (21) with $\sum_{r\in R_{w}}f_{rw}^{E}=d_{w}^{E}$ and summarize all OD pairs *w* ∈ *W*. Then, we multiply both sides of inequality (22) with $\sum_{r\in R_{w}}f_{rw}^{G}=d_{w}^{G}$ and summarize all *w* ∈ *W*. Finally, we sum these all and have:

$$\sum_{a\in A}\lambda_{a}^{E}v_{a}^{E}+\sum_{a\in A}\lambda_{a}^{G}v_{a}^{G}+\sum_{w\in W}\mu_{w}^{G}d_{w}^{G}+\sum_{w\in W}\mu_{w}^{E}d_{w}^{E}\leq \sum_{a\in A}e_{a}\left(v_{a}\right)v_{a}^{G}+\sum_{a\in A}t_{a}\left(v_{a}\right)v_{a}^{E}$$ (70)

Considering inequality (69), the LHS of inequality (70) can be written as:

$$\sum_{a\in A}e_{a}\left(v_{a}\right)v_{a}^{G}+P\sum_{a\in A}t_{a}\left(v_{a}\right)\cdot v_{a}\leq \sum_{a\in A}e_{a}\left(v_{a}\right)v_{a}^{G}+\sum_{w\in W}\mu_{w}^{E}d_{w}^{E}\text{+}\sum_{a\in A}\lambda_{a}^{E}v_{a}^{E}$$ (71)

By rewriting the right-hand side (RHS) of inequality (71) and combining with Eq (11), we have:

$$\sum_{a\in A}e_{a}\left(v_{a}\right)v_{a}^{G}+P\sum_{a\in A}t_{a}\left(v_{a}\right)\cdot v_{a}\leq \sum_{a\in A}\eta_{\text{max}}t_{a}\left(v_{a}\right)v_{a}+\sum_{a\in A}t_{a}\left(v_{a}\right)v_{a}-\sum_{a\in A}\frac{e_{a}\left(v_{a}\right)v_{a}^{G}}{\eta_{\text{max}}}$$ (72)

After simplifying inequality (72), we have:

$$\frac{E\left(v\right)}{T\left(v\right)}\leq \frac{\left(\eta_{\text{max}}+1-P\right)}{\left(1+1/\eta_{\text{max}}\right)}$$ (73)

By combining inequality (55) and inequality (73), we obtain Lemma 1.

Specifically, let **v** denote any feasible link flow patterns, and let **v**^*ME*^ and **v**^***SO***^ be the link flow pattern with the objective of minimum total system emissions *E*_*min*_ = 〈**v**^*ME*,^ *e*(**v**^*ME*^)〉 and minimum total system travel time *T*_*min*_ = 〈**v**^*SO*,^ *e*(**v**^*SO*^)〉.

For a given Pareto optimal flow, we are interested in how far the single system criterion *T*(**v**) or *E***(v**) could deviate from their minima, *T*_*min*_ and *E*_*min*_, respectively. To measure the deviation gap in this two-criteria system, we define two deviation factors:

$$\alpha_{T}\left(v\right)=\frac{T\left(v\right)}{T_{\text{min}}}=\frac{\langle v,t\left(v\right)\rangle }{\langle v^{SO},t\left(v^{SO}\right)\rangle }$$ (74)

$$\alpha_{E}\left(v\right)=\frac{E\left(v\right)}{E_{\text{min}}}=\frac{\langle v,e\left(v\right)\rangle }{\langle v^{ME},e\left(v^{ME}\right)\rangle }$$ (75)

where α_*T*_(**v**) measures the deviation gap between the total system travel time of any Pareto-efficient flow pattern *T*(**v**) and the minimum system travel time *T*_*min*_, and α_*E*_(**v**) measures the deviation gap between the total system emissions between any feasible flow pattern *E*(**v**) and the minimum system emissions *E*_*min*_.

For any Pareto optimal link flow **v**, $\alpha_{T}^{*}$ and $\alpha_{E}^{*}$ represent the upper bounds of α_*T*_(**v**) and α_*E*_(**v**), respectively; $\alpha_{T}(v)\leq \alpha_{T}^{*},\alpha_{E}(v)\leq \alpha_{E}^{*}.\alpha_{T}^{*}$ and $\alpha_{E}^{*}$ are defined in Eqs (76) and (77):

$$\alpha_{T}^{*}=\frac{T\left(v^{ME}\right)}{T_{\text{min}}}=\frac{T\left(v^{ME}\right)}{T\left(v^{SO}\right)}=\frac{\langle v^{ME},t\left(v^{ME}\right)\rangle }{\langle v^{SO},t\left(v^{SO}\right)\rangle }$$ (76)

$$\alpha_{{}_{E}}^{*}=\frac{E\left(v^{SO}\right)}{E_{\text{min}}}=\frac{E\left(v^{SO}\right)}{E\left(v^{ME}\right)}=\frac{\langle v^{SO},e\left(v^{SO}\right)\rangle }{\langle v^{ME},e\left(v^{ME}\right)\rangle }$$ (77)

After investigating the bound of $\alpha_{T}^{*}(v)$ and $\alpha_{E}^{*}(v)$, we obtain Lemma 1. Then, Proposition 3 can be easily obtained through Lemma 1.

### Appendix 3. Proof of Proposition 4

According to variation inequality (38), we have:

$$\langle t({\bar{v}}^{E}+{\bar{v}}^{G}),{\bar{v}}^{E}+{\bar{v}}^{G}\rangle \leq \langle t({\bar{v}}^{E}+{\bar{v}}^{G}),{\hat{v}}^{E}+{\hat{v}}^{G}\rangle$$ (78)

According to (60), we have:

$$\langle t({\bar{v}}^{E}+{\bar{v}}^{G}),{\bar{v}}^{E}+{\bar{v}}^{G}\rangle -\langle t({\bar{v}}^{E}+{\bar{v}}^{G}),{\hat{v}}^{E}+{\hat{v}}^{G}\rangle \leq 0$$ (79)

According to inequality (79), $T\left(\bar{v}\right)$ can also be expressed as:

$$T\left(\bar{v}\right)\leq \left(\langle t({\bar{v}}^{E}+{\bar{v}}^{G}),{\hat{v}}^{E}+{\hat{v}}^{G}\rangle -\langle t({\hat{v}}^{E}+{\hat{v}}^{G}),{\hat{v}}^{E}+{\hat{v}}^{G}\rangle \right)+\langle t({\hat{v}}^{E}+{\hat{v}}^{G}),{\hat{v}}^{E}+{\hat{v}}^{G}\rangle$$ (80)

To obtain the upper bound of inequality (80), we focus on:

$$\underset{v\geq 0}{\text{max}}\langle t({\bar{v}}^{E}+{\bar{v}}^{G}),{\hat{v}}^{E}+{\hat{v}}^{G}\rangle -\langle c(v^{E}+v^{G}),{\hat{v}}^{E}+{\hat{v}}^{G}\rangle$$ (81)

subject to constraints (21)-(25).

According to the KKT condition of program (81), (21)-(25) and BPR function, we have:

$${\hat{v}}_{}^{E}={\left(\beta +1\right)}^{-1/\beta }{\bar{v}}_{{}_{}}^{E}$$ (82)

$${\hat{v}}_{}^{G}={\left(\beta +1\right)}^{-1/\beta }{\bar{v}}_{{}_{}}^{G}$$ (83)

Moreover, inequality (78) can be rewritten as inequality (84):

$$\begin{array}{l} \langle t({\bar{v}}^{E}+{\bar{v}}^{G}),{\bar{v}}^{E}+{\bar{v}}^{G}\rangle -\left(\langle t({\bar{v}}^{E}+{\bar{v}}^{G}),{\hat{v}}^{E}+{\hat{v}}^{G}\rangle -\langle t({\hat{v}}^{E}+{\hat{v}}^{G}),{\hat{v}}^{E}+{\hat{v}}^{G}\rangle \right)\\ \leq \langle t({\hat{v}}^{E}+{\hat{v}}^{G}),{\hat{v}}^{E}+{\hat{v}}^{G}\rangle \end{array}$$ (84)

By substituting Eqs (82) and (83) into the LHS of inequality (84), we obtain:

$$\begin{array}{l} \langle t({\bar{v}}^{E}+{\bar{v}}^{G}),{\bar{v}}^{E}+{\bar{v}}^{G}\rangle -\langle {\left(\beta +1\right)}^{-\frac{1}{\beta }}\cdot \frac{\beta }{\beta +1}\cdot t_{a}^{0}\cdot \alpha {\left(\frac{{}_{v^{E}+v^{G}}}{c_{a}}\right)}^{\beta },{\bar{v}}^{E}+{\bar{v}}^{G}\rangle \\ \geq \langle t({\bar{v}}^{E}+{\bar{v}}^{G}),{\bar{v}}^{E}+{\bar{v}}^{G}\rangle -\langle {\left(\beta +1\right)}^{-\frac{1}{\beta }}\cdot \frac{\beta }{\beta +1}\cdot t_{a}^{0}\left[1+\alpha {\left(\frac{{}_{{\bar{v}}^{E}+{\bar{v}}^{G}}}{c_{a}}\right)}^{\beta }\right],{\bar{v}}^{E}+{\bar{v}}^{G}\rangle \\ =\left(1-{\left(\beta +1\right)}^{-\frac{1}{\beta }}\cdot \frac{\beta }{\beta +1}\right)\langle t({\bar{v}}^{E}+{\bar{v}}^{G}),{\bar{v}}^{E}+{\bar{v}}^{G}\rangle \end{array}$$ (85)

By combining inequality (84) and inequality (85), we have:

$$POA_{T}=\frac{\langle t({\bar{v}}^{E}+{\bar{v}}^{G}),{\bar{v}}^{E}+{\bar{v}}^{G}\rangle }{\langle t({\hat{v}}^{E}+{\hat{v}}^{G}),{\hat{v}}^{E}+{\hat{v}}^{G}\rangle }\leq 1/\left(1-{\left(\beta +1\right)}^{-\frac{1}{\beta }}\cdot \frac{\beta }{\beta +1}\right)$$ (86)

### Appendix 4. Proof of Proposition 5

**Proof.** It is well known that if a strategy distribution is a user equilibrium, then it satisfies variational inequality (87):

$$\langle t\left({\bar{v}}^{E}+{\bar{v}}^{G}\right),{\hat{v}}^{E}+{\hat{v}}^{G}-\left({\bar{v}}^{E}+{\bar{v}}^{G}\right)\rangle \geq 0$$ (87)

Inequality (87) can be rewritten as:

$$\langle t({\bar{v}}^{E}+{\bar{v}}^{G}),{\bar{v}}^{E}+{\bar{v}}^{G}\rangle \leq \langle t({\bar{v}}^{E}+{\bar{v}}^{G}),{\hat{v}}^{E}+{\hat{v}}^{G}\rangle$$ (88)

The link-based aggregate link flow without tolls $\bar{v}$ and aggregate link flow under differentiable tolls can be expresses in (89) and (90):

$$\bar{v}={\bar{v}}^{E}+{\bar{v}}^{G}$$ (89)

$$\hat{v}={\hat{v}}^{E}+{\hat{v}}^{G}$$ (90)

Similarly, the BPR time function is used, in which the capacity *C*_*a*_ is set as 1 and free flow time *t*_0_ is set as 0 to simplify the results. Then inequality (88) can be rewritten as inequality (91):

$$\langle t(\bar{v}),{\bar{v}}^{G}\rangle \leq \langle t(\hat{v}),{\hat{v}}^{G}\rangle +\langle t(\bar{v}),{\hat{v}}^{E}-{\bar{v}}^{E}\rangle +\langle t(\bar{v})-t(\hat{v}),{\hat{v}}^{G}\rangle +\langle \alpha {\bar{v}}^{\beta },\hat{v}-\bar{v}\rangle$$ (91)

And then inequality (91) can be rewritten as inequality (92):

$$\langle t(\bar{v}),{\bar{v}}^{G}\rangle \leq \langle t(\hat{v}),v^{G}\rangle +\langle t(\bar{v})-t(\hat{v}),v^{G}\rangle +\langle \alpha {\bar{v}}^{\beta },\hat{v}-\bar{v}\rangle$$ (92)

**Fig 13:**
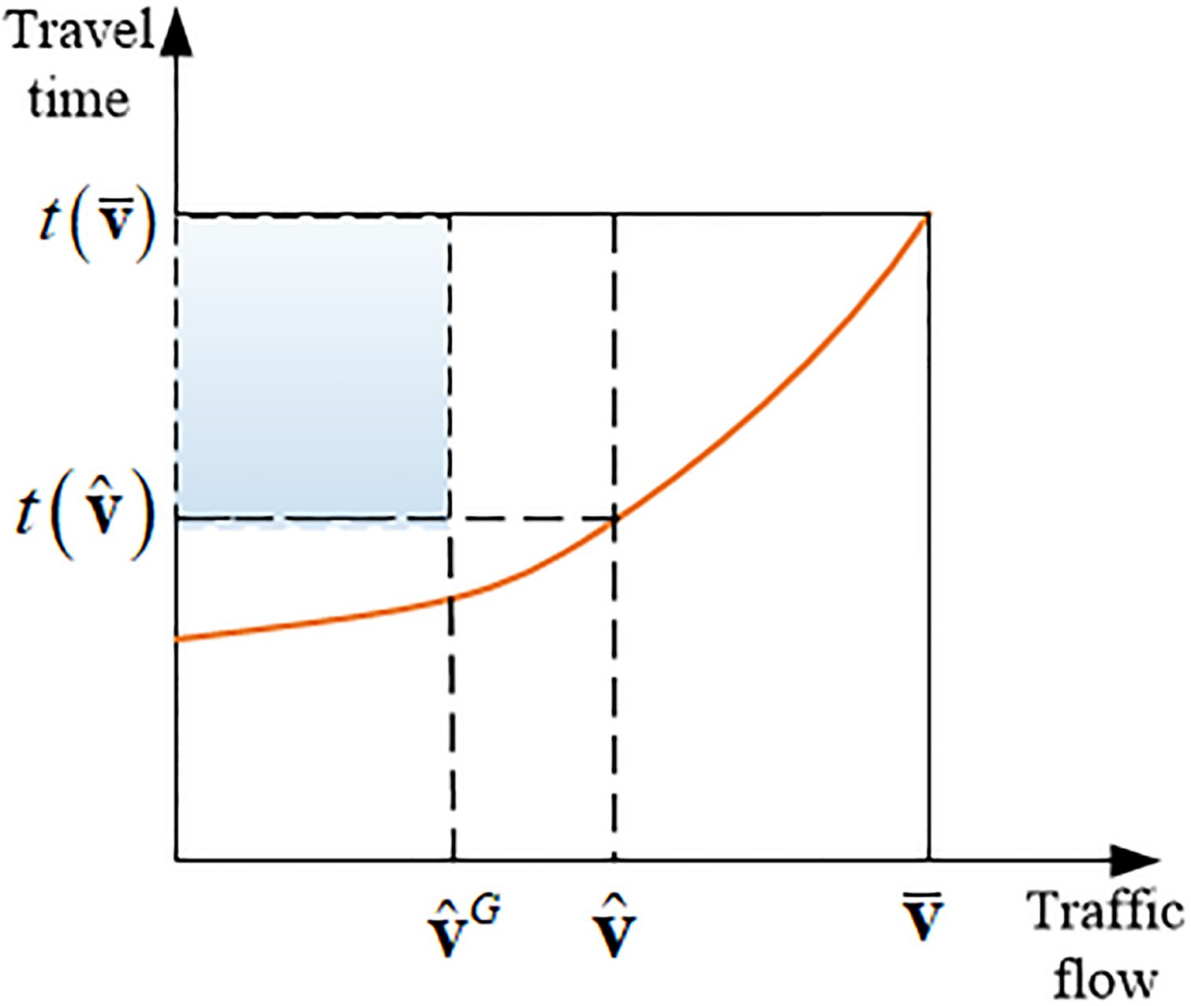
Geometric illustration of the definition of *γ*_*a*_.

We now consider the case with nonlinear separable link travel time function, see Fig 13, this geometric approach to the price of anarchy is first proposed and illustrated in [39].

In order to upper bound the shaded area in Fig 13, or the term $\langle t(\bar{v})-t(v),v^{G}\rangle$, the following parameter *γ*_*a*_ is defined in (1)-(4):

$$\gamma_{a}(T,\bar{v},\hat{v},{\bar{\tau }}_{a},\beta )=\underset{t_{a}\in T,a\in A}{\text{sup}}\gamma_{a}(T,\bar{v},\hat{v},{\bar{\tau }}_{a},\beta )$$ (93)

According to inequality (92), the parameter *γ*_*a*_ can be reformulated as following:

$$\gamma_{a}(T,\bar{v},\hat{v},{\bar{\tau }}_{a},\beta )=\underset{x,x^{G}\geq 0}{\text{max}}\frac{\langle t(\bar{v})-t(\hat{v}),{\hat{v}}^{G}\rangle +\langle \alpha {\bar{v}}^{\beta },\hat{v}-\bar{v}\rangle }{\langle t(\bar{v}),{\bar{v}}^{G}\rangle }$$ (94)

subject to constraints (1)-(4), (94).

Here, 0/0 by convention. According to the KKT condition of program (21)-(25), we can obtain that:

$${\hat{v}}_{a}={\left(\beta +1\right)}^{-1/\beta }{\bar{v}}_{a}$$ (95)

And thus, we have:

$$\gamma_{a}(T,\bar{v},\hat{v},{\bar{\tau }}_{a},\beta )=\underset{a\in A}{\text{max}}\frac{\left(t({\bar{v}}_{a})-t({\hat{v}}_{a})\right)\cdot {\hat{v}}_{a}^{G}+\alpha {\bar{v}}^{\beta }\cdot \left({\hat{v}}_{a}-{\bar{v}}_{a}\right)}{t_{a}({\bar{v}}_{a}){\bar{v}}_{a}^{G}}$$ (96)

$$\leq \frac{\left[\alpha {\left({\bar{v}}_{a}\right)}^{\beta }-\alpha {\left(\beta +1\right)}^{-1}{\bar{v}}_{a}{}^{\beta }\right]\cdot {\left(\beta +1\right)}^{-1/\beta }{\bar{v}}_{a}+\alpha {\bar{v}}^{\beta }\cdot \left({\left(\beta +1\right)}^{-1/\beta }-1\right)\cdot {\bar{v}}_{a}}{\alpha {\left({\bar{v}}_{a}\right)}^{\beta +1}}$$ (97)

$$\leq {\left(\beta +1\right)}^{-1/\beta }-{\left(\beta +1\right)}^{-1}$$ (98)

According to inequality (92), we have:

$$\langle t(\bar{v}),{\bar{v}}^{G}\rangle \leq \langle t(\hat{v}),{\hat{v}}^{G}\rangle +\gamma_{a}\langle t(\bar{v}),{\bar{v}}^{G}\rangle$$ (99)

And thus, inequality can be rewritten as inequality

$$\frac{\langle t(\bar{v}),{\bar{v}}^{G}\rangle }{\langle t(\hat{v}),{\hat{v}}^{G}\rangle }\leq \frac{1}{1-\gamma_{a}}$$ (100)

According to the relationship between emission factor *η*_*a*_(*v*_*a*_) and link time function, the inequality (100) can be rewritten as inequality (101):

$$\frac{\bar{E}}{\hat{E}}\leq \frac{\eta_{\text{max}}\langle t(\bar{v}),{\bar{v}}^{G}\rangle }{\eta_{\text{min}}\langle t(\hat{v}),{\hat{v}}^{G}\rangle }\leq \frac{\eta_{\text{max}}}{\eta_{\text{min}}[1-\gamma_{a}(T,\bar{v},\hat{v},{\bar{\tau }}_{a},\beta )]}$$ (101)

According to the definition of the price of anarchy for total system emissions *POA*_*E*_ given in (62), we have:

$$POA_{E}\leq \frac{\eta_{\text{max}}}{\eta_{\text{min}}\left[1-{\left(\beta +1\right)}^{-1/\beta }-{\left(\beta +1\right)}^{-1}\right]}$$ (102)

Thus, Proposition 5 is obtained.

## Supporting information

S1 Data Results of the total system travel time and total system emissions in Shenzhen. (CSV)

S2 Data Results of the total system travel time and total system emissions in Lasa. (CSV)

S3 Data Numerical results for the *POA*_*E*_ in Shenzhen. (DOCX)

S4 Data Numerical results for the *POA*_*E*_ in Lasa. (DOCX)

S1 Text C++ Codes for second development on Trans CAD. (TXT)

S2 Text Input OD table for Shenzhen. (TXT)

S1 File Map information. (ZIP)

S2 File Shenzhen OD Data. (ZIP)

S3 File Street information. (ZIP)

S1 Fig (JPG)
