# Supplementary material for: Bounding the efficiency gain of differentiable road pricing for EVs and GVs to manage congestion and emissions
Source: PLoS One. 2020 Jul 30;15(7):e0234204. doi: 10.1371/journal.pone.0234204 (PMC7392306; doi:10.1371/journal.pone.0234204)
Supplement: S3 Data — (DOCX) [file pone.0234204.s003.docx]

| 1 | 2 | 3 | 4 | 5 | 6 | 7 | 8 | 9 | 10 |
| --- | --- | --- | --- | --- | --- | --- | --- | --- | --- |
| Luo hu | Fu tian | Nan Shan | Yan  tian | Bao  an | Long gang | Guang  ming | Ping shan | Long hua | Da peng |

Table S2.1 the value of ${POA}_{E}$ in district 1 of Shenzhen

| *P* | 0.5 | 0.75 | 1 | $\Phi$  1.25 | 1.5 | 1.75 | 2 |
| --- | --- | --- | --- | --- | --- | --- | --- |
| 0 | 1.064409 | 1.089549 | 1.112432 | 1.43715 | 1.165629 | 1.183169 | 1.19411 |
| 0.1 | 1.064504 | 1.089502 | 1.112457 | 1.437009 | 1.165573 | 1.183138 | 1.194031 |
| 0.2 | 1.06439 | 1.089582 | 1.112457 | 1.437096 | 1.165622 | 1.183173 | 1.194029 |
| 0.3 | 1.06452 | 1.089631 | 1.112592 | 1.437074 | 1.165579 | 1.183129 | 1.194127 |
| 0.4 | 1.064728 | 1.08966 | 1.112616 | 1.437303 | 1.165702 | 1.183182 | 1.194074 |
| 0.5 | 1.064617 | 1.089781 | 1.112656 | 1.43738 | 1.165752 | 1.183266 | 1.194301 |
| 0.6 | 1.064858 | 1.090068 | 1.112907 | 1.43782 | 1.165935 | 1.183387 | 1.194242 |
| 0.7 | 1.064975 | 1.090387 | 1.113201 | 1.438138 | 1.16607 | 1.1835 | 1.194422 |
| 0.8 | 1.065903 | 1.090957 | 1.113386 | 1.439058 | 1.166648 | 1.184046 | 1.194596 |
| 0.9 | 1.066184 | 1.092601 | 1.115076 | 1.441422 | 1.167597 | 1.185025 | 1.195581 |

Table S2.2 the value of ${POA}_{E}$ in district 2 of Shenzhen

| *P* | 0.5 | 0.75 | 1 | $\Phi$  1.25 | 1.5 | 1.75 | 2 |
| --- | --- | --- | --- | --- | --- | --- | --- |
| 0 | 1.042764 | 1.075542 | 1.104442 | 1.436415 | 1.156083 | 1.172067 | 1.184219 |
| 0.1 | 1.042612 | 1.075502 | 1.104372 | 1.436333 | 1.155993 | 1.172033 | 1.184183 |
| 0.2 | 1.042644 | 1.075546 | 1.104285 | 1.436389 | 1.156099 | 1.172102 | 1.184268 |
| 0.3 | 1.042562 | 1.075502 | 1.104403 | 1.436475 | 1.156055 | 1.172136 | 1.184231 |
| 0.4 | 1.042821 | 1.075532 | 1.104484 | 1.43657 | 1.15616 | 1.172183 | 1.184324 |
| 0.5 | 1.043024 | 1.075761 | 1.104675 | 1.436784 | 1.156239 | 1.172185 | 1.18428 |
| 0.6 | 1.043053 | 1.075945 | 1.104676 | 1.437027 | 1.15633 | 1.172338 | 1.18446 |
| 0.7 | 1.043259 | 1.076 | 1.104871 | 1.437228 | 1.156439 | 1.172426 | 1.184508 |
| 0.8 | 1.044055 | 1.076641 | 1.105292 | 1.438119 | 1.156729 | 1.172886 | 1.184936 |
| 0.9 | 1.045119 | 1.077698 | 1.106733 | 1.440667 | 1.157717 | 1.173367 | 1.185529 |

Table S2.3 the value of ${POA}_{E}$ in district 3 of Shenzhen

| *P* | 0.5 | 0.75 | 1 | $\Phi$  1.25 | 1.5 | 1.75 | 2 |
| --- | --- | --- | --- | --- | --- | --- | --- |
| 0 | 1.037208 | 1.072614 | 1.104847 | 1.400079 | 1.151692 | 1.166688 | 1.188307 |
| 0.1 | 1.037118 | 1.072604 | 1.104752 | 1.399956 | 1.151635 | 1.166582 | 1.188174 |
| 0.2 | 1.037314 | 1.072533 | 1.104751 | 1.40002 | 1.151665 | 1.166669 | 1.188226 |
| 0.3 | 1.03728 | 1.072526 | 1.104881 | 1.39999 | 1.151622 | 1.166676 | 1.188202 |
| 0.4 | 1.0372 | 1.072637 | 1.104805 | 1.400245 | 1.151813 | 1.166713 | 1.188271 |
| 0.5 | 1.037343 | 1.072955 | 1.10503 | 1.400475 | 1.151904 | 1.166724 | 1.188331 |
| 0.6 | 1.037766 | 1.072887 | 1.10507 | 1.400705 | 1.15194 | 1.16688 | 1.188294 |
| 0.7 | 1.037488 | 1.073084 | 1.105449 | 1.401143 | 1.152135 | 1.167034 | 1.188661 |
| 0.8 | 1.038295 | 1.073548 | 1.105746 | 1.402048 | 1.152677 | 1.167383 | 1.188923 |
| 0.9 | 1.038291 | 1.075179 | 1.107137 | 1.404736 | 1.153887 | 1.168088 | 1.189765 |

Table S2.4 the value of ${POA}_{E}$ in district 4 of Shenzhen

| *P* | 0.5 | 0.75 | 1 | $\Phi$  1.25 | 1.5 | 1.75 | 2 |
| --- | --- | --- | --- | --- | --- | --- | --- |
| 0 | 1.0202 | 1.0089 | 1.0093 | 1.2286 | 1.0091 | 1.0285 | 1.0396 |
| 0.1 | 1.0201 | 1.0091 | 1.0093 | 1.2284 | 1.0091 | 1.0284 | 1.0396 |
| 0.2 | 1.0202 | 1.0091 | 1.0093 | 1.2286 | 1.0090 | 1.0283 | 1.0395 |
| 0.3 | 1.0204 | 1.0091 | 1.0093 | 1.2284 | 1.0090 | 1.0286 | 1.0394 |
| 0.4 | 1.0203 | 1.0091 | 1.0093 | 1.2287 | 1.0091 | 1.0286 | 1.0395 |
| 0.5 | 1.0202 | 1.0091 | 1.0093 | 1.2288 | 1.0091 | 1.0284 | 1.0395 |
| 0.6 | 1.0207 | 1.0090 | 1.0093 | 1.2288 | 1.0092 | 1.0283 | 1.0395 |
| 0.7 | 1.0206 | 1.0090 | 1.0093 | 1.2288 | 1.0093 | 1.0292 | 1.0398 |
| 0.8 | 1.0212 | 1.0090 | 1.0093 | 1.2298 | 1.0097 | 1.0283 | 1.0401 |
| 0.9 | 1.0217 | 1.0090 | 1.0094 | 1.2317 | 1.0098 | 1.0289 | 1.0409 |

Table S2.5 the value of ${POA}_{E}$ in district 5 of Shenzhen

| *P* | 0.5 | 0.75 | 1 | $\Phi$  1.25 | 1.5 | 1.75 | 2 |
| --- | --- | --- | --- | --- | --- | --- | --- |
| 0 | 1.054005 | 1.078729 | 1.098511 | 1.369943 | 1.130715 | 1.146465 | 1.159512 |
| 0.1 | 1.054003 | 1.078726 | 1.098382 | 1.36988 | 1.130648 | 1.146343 | 1.159397 |
| 0.2 | 1.054144 | 1.078735 | 1.098469 | 1.369924 | 1.130658 | 1.146316 | 1.159393 |
| 0.3 | 1.054085 | 1.078757 | 1.098485 | 1.370061 | 1.130752 | 1.146436 | 1.159488 |

| 0.4 | 1.054269 | 1.078881 | 1.09861 | 1.370189 | 1.130777 | 1.146464 | 1.159538 |
| --- | --- | --- | --- | --- | --- | --- | --- |
| 0.5 | 1.054216 | 1.078964 | 1.098719 | 1.370339 | 1.130861 | 1.146544 | 1.159606 |
| 0.6 | 1.054636 | 1.07906 | 1.0988 | 1.370644 | 1.131021 | 1.14662 | 1.159652 |
| 0.7 | 1.054535 | 1.079464 | 1.099117 | 1.371129 | 1.131163 | 1.14691 | 1.159875 |
| 0.8 | 1.055622 | 1.080043 | 1.099562 | 1.372327 | 1.131685 | 1.147115 | 1.160124 |
| 0.9 | 1.057057 | 1.081489 | 1.101052 | 1.375078 | 1.132951 | 1.148404 | 1.161217 |

Table S2.6 the value of ${POA}_{E}$ in district 6 of Shenzhen

| *P* | 0.5 | 0.75 | 1 | $\Phi$  1.25 | 1.5 | 1.75 | 2 |
| --- | --- | --- | --- | --- | --- | --- | --- |
| 0 | 1.041986 | 1.061401 | 1.078409 | 1.358536 | 1.103722 | 1.119171 | 1.139815 |
| 0.1 | 1.041932 | 1.061341 | 1.078288 | 1.358369 | 1.103602 | 1.119042 | 1.139735 |
| 0.2 | 1.041992 | 1.061361 | 1.078298 | 1.358384 | 1.103636 | 1.119105 | 1.13976 |
| 0.3 | 1.042083 | 1.061493 | 1.078388 | 1.358568 | 1.103689 | 1.119115 | 1.139797 |
| 0.4 | 1.042023 | 1.061527 | 1.078414 | 1.358656 | 1.1037 | 1.1192 | 1.139828 |
| 0.5 | 1.041975 | 1.061591 | 1.078491 | 1.358885 | 1.103832 | 1.119268 | 1.13981 |
| 0.6 | 1.042431 | 1.06175 | 1.078605 | 1.359009 | 1.103897 | 1.119354 | 1.139982 |
| 0.7 | 1.042315 | 1.061843 | 1.078805 | 1.359558 | 1.104049 | 1.119408 | 1.140115 |
| 0.8 | 1.04299 | 1.062266 | 1.079214 | 1.360313 | 1.104435 | 1.119919 | 1.140479 |
| 0.9 | 1.0441 | 1.062951 | 1.080032 | 1.362779 | 1.105305 | 1.120688 | 1.141348 |

Table S2.7 the value of ${POA}_{E}$ in district 7 of Shenzhen

| P | 0.5 | 0.75 | 1 | $\Phi$  1.25 | 1.5 | 1.75 | 2 |
| --- | --- | --- | --- | --- | --- | --- | --- |
| 0 | 1.00268 | 1.007874 | 1.009913 | 1.266525 | 1.046665 | 1.057169 | 1.067478 |
| 0.1 | 1.002666 | 1.00778 | 1.009843 | 1.266354 | 1.046488 | 1.057052 | 1.067433 |
| 0.2 | 1.002783 | 1.00784 | 1.009965 | 1.266374 | 1.04653 | 1.057078 | 1.067445 |
| 0.3 | 1.002745 | 1.007918 | 1.010001 | 1.266525 | 1.046638 | 1.057219 | 1.067365 |
| 0.4 | 1.0027 | 1.00788 | 1.010014 | 1.266615 | 1.04657 | 1.057128 | 1.067544 |
| 0.5 | 1.002894 | 1.007597 | 1.010052 | 1.267013 | 1.046719 | 1.057358 | 1.067566 |
| 0.6 | 1.002859 | 1.008066 | 1.010163 | 1.266865 | 1.046695 | 1.057107 | 1.067658 |
| 0.7 | 1.002878 | 1.008011 | 1.01035 | 1.267144 | 1.046526 | 1.057393 | 1.067703 |
| 0.8 | 1.002688 | 1.007611 | 1.010292 | 1.268155 | 1.04699 | 1.057491 | 1.068037 |
| 0.9 | 1.004136 | 1.007835 | 1.011422 | 1.270617 | 1.046997 | 1.058455 | 1.0685 |

Table S2.8 the value of ${POA}_{E}$ in district 8 of Shenzhen

| *P* | 0.5 | 0.75 | 1 | $\Phi$  1.25 | 1.5 | 1.75 | 2 |
| --- | --- | --- | --- | --- | --- | --- | --- |
| 0 | 1.008926 | 1.02077 | 1.041665 | 1.326341 | 1.073903 | 1.088439 | 1.099018 |
| 0.1 | 1.009448 | 1.020804 | 1.041729 | 1.325894 | 1.073532 | 1.088195 | 1.099265 |
| 0.2 | 1.009633 | 1.019975 | 1.041341 | 1.326793 | 1.073897 | 1.088035 | 1.099055 |
| 0.3 | 1.008883 | 1.020831 | 1.041457 | 1.326155 | 1.073536 | 1.08816 | 1.0994 |
| 0.4 | 1.008825 | 1.02069 | 1.041801 | 1.326666 | 1.073972 | 1.088208 | 1.099605 |
| 0.5 | 1.008871 | 1.020798 | 1.041385 | 1.326903 | 1.073963 | 1.088838 | 1.099212 |
| 0.6 | 1.009092 | 1.019746 | 1.041327 | 1.327357 | 1.074343 | 1.088517 | 1.099248 |
| 0.7 | 1.008612 | 1.02141 | 1.041558 | 1.32763 | 1.074522 | 1.089239 | 1.1002 |
| 0.8 | 1.008934 | 1.020829 | 1.043123 | 1.32946 | 1.074566 | 1.08907 | 1.09994 |
| 0.9 | 1.012541 | 1.024545 | 1.043478 | 1.331665 | 1.074808 | 1.092234 | 1.102304 |

Table S2.9 the value of ${POA}_{E}$ in district 9 of Shenzhen

| *P* | 0.5 | 0.75 | 1 | $\Phi$  1.25 | 1.5 | 1.75 | 2 |
| --- | --- | --- | --- | --- | --- | --- | --- |
| 0 | 1.020214 | 1.024676 | 1.041433 | 1.317707 | 1.074358 | 1.090491 | 1.106122 |
| 0.1 | 1.020146 | 1.024482 | 1.041377 | 1.317657 | 1.074273 | 1.090446 | 1.106007 |
| 0.2 | 1.020155 | 1.0246 | 1.041444 | 1.317632 | 1.074293 | 1.090439 | 1.106064 |
| 0.3 | 1.020384 | 1.024639 | 1.041419 | 1.31766 | 1.074274 | 1.090471 | 1.106096 |
| 0.4 | 1.020342 | 1.024618 | 1.041451 | 1.317688 | 1.074367 | 1.090472 | 1.106096 |
| 0.5 | 1.020218 | 1.024673 | 1.041599 | 1.317811 | 1.074335 | 1.090536 | 1.106188 |
| 0.6 | 1.020719 | 1.024854 | 1.041735 | 1.317813 | 1.074511 | 1.090596 | 1.106203 |
| 0.7 | 1.020561 | 1.024861 | 1.041654 | 1.317848 | 1.07462 | 1.090685 | 1.106268 |
| 0.8 | 1.021187 | 1.025231 | 1.042243 | 1.318141 | 1.07476 | 1.09101 | 1.106553 |
| 0.9 | 1.021744 | 1.026723 | 1.042606 | 1.318882 | 1.075128 | 1.091864 | 1.106862 |

Table S2.10 the value of ${POA}_{E}$ in district 10 of Shenzhen

| *`P* | 0.5 | 0.75 | 1 | $\Phi$  1.25 | 1.5 | 1.75 | 2 |
| --- | --- | --- | --- | --- | --- | --- | --- |
| 0 | 1.00795 | 1.013193 | 1.017791 | 1.241495 | 1.026564 | 1.037141 | 1.046967 |
| 0.1 | 1.007661 | 1.013324 | 1.017586 | 1.241315 | 1.026333 | 1.037088 | 1.046814 |
| 0.2 | 1.008539 | 1.013052 | 1.017768 | 1.241339 | 1.026274 | 1.037081 | 1.047041 |
| 0.3 | 1.008359 | 1.013263 | 1.017747 | 1.241554 | 1.0266 | 1.037192 | 1.047013 |
| 0.4 | 1.008759 | 1.014282 | 1.01826 | 1.242107 | 1.026802 | 1.037201 | 1.047123 |
| 0.5 | 1.008232 | 1.013339 | 1.017887 | 1.242587 | 1.026941 | 1.037392 | 1.046853 |
| 0.6 | 1.007832 | 1.013307 | 1.019252 | 1.241898 | 1.026916 | 1.037043 | 1.047016 |
| 0.7 | 1.00768 | 1.014388 | 1.017881 | 1.243648 | 1.027573 | 1.037233 | 1.047125 |
| 0.8 | 1.009716 | 1.01356 | 1.021129 | 1.243004 | 1.027378 | 1.037177 | 1.04768 |
| 0.9 | 1.011218 | 1.013554 | 1.02223 | 1.250652 | 1.030072 | 1.036605 | 1.04668 |
