# Supplementary material for: Bounding the efficiency gain of differentiable road pricing for EVs and GVs to manage congestion and emissions
Source: PLoS One. 2020 Jul 30;15(7):e0234204. doi: 10.1371/journal.pone.0234204 (PMC7392306; doi:10.1371/journal.pone.0234204)
Supplement: S4 Data — (DOCX) [file pone.0234204.s004.docx]

| *P* | 0.5 | 0.75 | 1 | $\Phi$  1.25 | 1.5 | 1.75 | 2 |
| --- | --- | --- | --- | --- | --- | --- | --- |
| 0 | 1.0420 | 1.0614 | 1.0784 | 1.2643 | 1.1037 | 1.1192 | 1.1398 |
| 0.1 | 1.0419 | 1.0613 | 1.0782 | 1.2543 | 1.1036 | 1.1190 | 1.1397 |
| 0.2 | 1.0420 | 1.0614 | 1.0782 | 1.2643 | 1.1036 | 1.1192 | 1.1399 |
| 0.3 | 1.0421 | 1.0615 | 1.0783 | 1.2677 | 1.1039 | 1.1191 | 1.1397 |
| 0.4 | 1.0420 | 1.0615 | 1.0784 | 1.2653 | 1.1037 | 1.1192 | 1.1396 |
| 0.5 | 1.0420 | 1.0616 | 1.0785 | 1.2643 | 1.1038 | 1.1195 | 1.1398 |
| 0.6 | 1.0424 | 1.0618 | 1.0788 | 1.2633 | 1.1039 | 1.1193 | 1.1346 |
| 0.7 | 1.0423 | 1.06065 | 1.0788 | 1.2645 | 1.1040 | 1.1196 | 1.1401 |
| 0.8 | 1.0423 | 1.0623 | 1.0792 | 1.2665 | 1.1044 | 1.1199 | 1.1404 |
| 0.9 | 1.0441 | 1.0634 | 1.0800 | 1.2678 | 1.1053 | 1.1207 | 1.1413 |

Numerical results of ${POA}_{E}$in Lasa
