# Supplementary figures and images for: Bounding the efficiency gain of differentiable road pricing for EVs and GVs to manage congestion and emissions
Source: PLoS One. 2020 Jul 30;15(7):e0234204. doi: 10.1371/journal.pone.0234204 (PMC7392306; doi:10.1371/journal.pone.0234204)

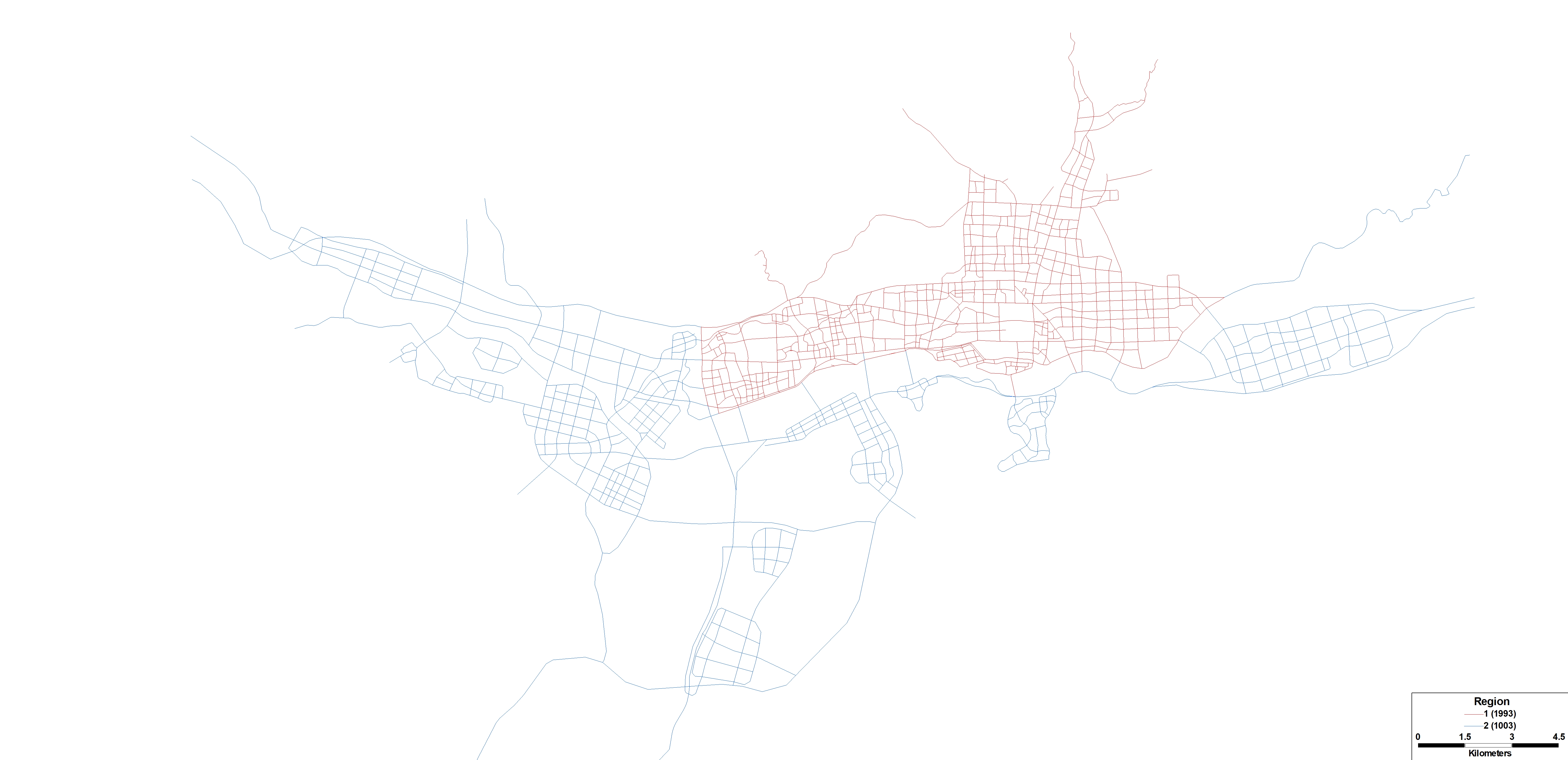

Supplement: S1 Fig — (JPG) [file pone.0234204.s010.jpg]
